# Computationally Efficient DFT-Based Sampling of Ion Diffusion in Crystalline Solids

**DOI:** 10.1021/acs.jctc.5c00891

**Published:** 2025-09-03

**Authors:** Hannes Gustafsson, Fabian Schwarz, Thijs Smolders, Senja Barthel, Amber Mace

**Affiliations:** † Department of Chemistry − Ångström, 8097 Uppsala University, SE-751 21 Uppsala, Sweden; ‡ Department of Mathematics, 1190 Vrije Universiteit Amsterdam, 1081 HV Amsterdam, Netherlands

## Abstract

We present a method for large-scale DFT-based screening of ion diffusion in crystalline solids. This is accomplished by extending the Ionic TuTraSt method to sample the potential energy surface by using single-point DFT calculations. To drastically reduce the number of single-point DFT calculations, symmetry, interpolation, and exclusion of high-energy regions are employed. This approach is tested on a large data set of solid-state Li-ion conductors, for which the interpolation and high-energy exclusion are optimized to balance computational efficiency and accuracy of the obtained diffusion properties. Furthermore, the developed workflow is validated by comparison with ab initio molecular dynamics (AIMD) simulations on a set of known Li-ion superconducting materials.

## Graphical abstract

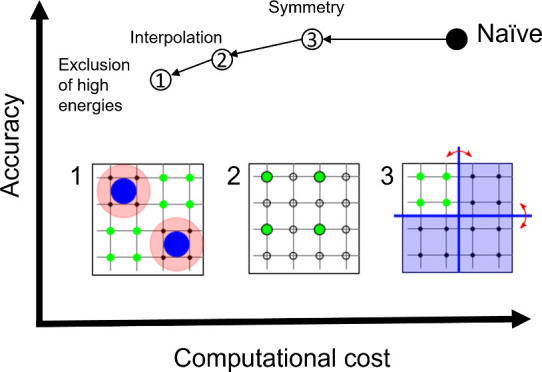

## 1 Introduction

Methods for sampling the configurational space-dependent potential energy surface (PES) are often a central part in the application of atomistic computational approaches that rely on statistical thermodynamics for property predictions.^1^ The standard approach involved in state-of-the-art molecular dynamics (MD) and Metropolis Monte Carlo (MMC) algorithms is Boltzmann sampling, where the probability of a specific configurational state to be sampled is related to the energy of that state according to the Boltzmann distribution law. However, this approach entails that rare event transition states are poorly sampled or not sampled at all within the time scale of a simulation. To overcome such challenges, guided enhanced sampling methods such as metadynamics and umbrella sampling, or minimum energy pathway methods like nudged elastic band (NEB),^2^ are often employed to provide a bias to the sampling toward the important high energy states. These methods, however, require prior knowledge of the important regions of the PES.

Another approach is to sample along configuration coordinates that are regularly arranged on a rectilinear grid. Such grid-based sampling has the benefit of providing unbiased, systematic, and uniform sampling and is particularly useful when the entire part of a configurational space is to be sampled or when it is difficult to determine a priori which regions of the PES are relevant. The uniform and complete sampling provided by a grid-based approach also brings benefits for analyzing topological and geometric features of the PES to predict materials properties.

For example, methods that make use of a three-dimensional representation of the PES have been widely and successfully applied to study guest particle adsorption and transport in nanoporous solids (e.g., Zeolites, MOFs, and COFs), with key applications such as gas separation. Such methods have enabled efficient calculations of Henry’s constants and adsorption energies^3^ and of diffusion barriers and channel estimates through energy sampling combined with a geometric analysis.^3–6^ In a similar manner, grid-based geometric and topological analysis to extract minimal energy paths, transition rates, and diffusion channels have been successfully developed for predicting ion transport in e.g., ceramic solid state electrolytes,^7–10^ including our previous work on the Ionic TuTraSt method.^11^

The Ionic TuTraSt method is a computational workflow that constructs the three-dimensional PES grid of the diffusing Li-ion within a rigid crystalline framework, followed by a MMC based correction to include the important electrostatic interactions between the mobile ions. A topological and geometrical analysis then follows on the full space of this constructed multiparticle PES grid which partitions it into energy minima basin volumes and transition state surfaces dividing them.^4^ By applying transition state theory, this information is then reduced to a lattice-based kinetic Monte Carlo (kMC) model^12^ for efficient computation of the ion self-diffusion coefficient.

One benefit of this method is that it maps out the full diffusion network in an automated approach without the need for beforehand knowledge of the PES, such as location of energy minima and transition states, or direction of transitions such as is needed for, e.g., NEB or other guided sampling based approaches to compute energy barriers for quantifying diffusion.^13–16^ This brings significant advantages for carrying out high-throughput screenings to predict diffusion properties throughout the diverse chemical space of ceramic electrolyte materials. Another benefit compared to minimum energy path methods such as NEB and SoftBV that compute only single point energy barriers, i.e., the difference between minimum and maximum energy for each defined transition, is that Ionic TuTraSt integrates and makes use of the full three-dimensional PES space and thus provides improved accuracy of the resulting transition rates which include the full configurational entropy of mobile ions within the (rigid) framework.

On the other hand, the main drawback of this grid-based approach is the number of explicit energy calculations required to construct the single particle input grid, which depends on the size of the periodic unit cell of the crystalline framework and the grid resolution required to ensure accuracy. This number generally lands in the range of tens to hundreds-of-thousands: For example, a grid resolution of 0.2 Å would for a 8 × 8 × 8 Å cubic unit cell involve 64 000 grid point calculations. This is several orders of magnitude lower than the number of energy calculations carried out in a typical MD simulation as shown in our previous work;^4,11^ however, it is many orders of magnitude higher than what would be needed for a, e.g., NEB-based approach, which in turn is less automatable. The number of grid energy calculations can be manageable for screening through a large number of materials using preparameterized force fields. However, the chemical diversity of solid-state inorganic ion conductors^17^ presents a challenge both for classical and machine learning force-fields. While accurate such force-fields exist and can be constructed for specific structures or families of compounds, they often require extensive parametrization to DFT-based calculations and generally lack the transferability which is crucial for exploring vastly varying chemical compositions. On the other hand, so-called universal force-fields such as UFF^18^ and the SoftBV force-field^9^ are instead parametrized for a very broad range of compositions but fall short in their precision compared to first-principles methods. While direct calculations of DFT-based single particle grids would offer significant improvements to both the accuracy and transferability, the comparably high computational cost of each energy calculation sets a bottleneck for high-throughput applications.

To address this challenge, this work presents strategies to reduce the number of explicit grid point energy calculations required to construct DFT-based PES grids, thereby lowering computational costs and enabling high-throughput applications. The grid-based structure permits several tools to be leveraged for this purpose. Interpolation of potential energy may be used to relax the resolution, allowing to reduce the sampling necessary for constructing the grid. The estimation, either by geometric or energetic means, of high energy regions such as volume blocked by the framework makes it possible to avoid the computational cost of sampling grid coordinates, where the ion is very improbable to reside. Lastly, the often high symmetry of crystalline inorganic materials makes the application of symmetry to reduce sampling highly beneficial, and rectilinear grids are well suited for this. In the current work, we employ these strategies in the sampling of single-particle DFT PES grids for the application of the Ionic TuTraSt method. We investigate the plausible computational savings, as well as the effect on diffusion predictions, and explore the limitations of the strategies. It is shown that, combined, these tools can reduce the grid points that need to be sampled by 2–3 orders of magnitude for the vast majority of structures, with only marginal loss of accuracy.

To benchmark the diffusion predictions from Ionic TuTraSt based on DFT grids, we compare our results with results from equivalent ab initio molecular dynamics (AIMD) for a set of Li-ion solid electrolytes with previously reported high Li-ion conductivities, for which it is computationally feasible to adequately reach the diffusive time scale in AIMD. Our results prove that reduction strategies available for grid-based sampling methods can enable fast, DFT-based diffusion predictions for ions in the solid state at extended time scales and in a high-throughput setting, with accuracy comparable to AIMD. This opens up a new avenue for efficient predictions of ion diffusivity in an expanded chemical space of crystalline inorganic materials.

## 2 Theory

The following subsections briefly introduce and discuss the three strategies used and evaluated in this work to reduce the number of required energy calculations when sampling a guest ion on a regular, rectilinear grid within a crystal framework. The first strategy makes use of space-group symmetry to eliminate redundant calculations. The second avoids sampling grid points that are blocked or associated with high energies. The third strategy applies grid-based interpolation to estimate energies at unsampled points.

### 2.1 Symmetry

The symmetries of three-dimensional periodic structures are described by the 230 different space groups resulting from all possible combinations of point group symmetries and Bravais lattices that describe the periodicity in three dimensions. Information about the symmetry of a structure can be used since it can greatly reduce the workload of a calculation without losing any accuracy. When considering the potential energy *E*(***r***) of a single guest particle inside a periodic framework structure, the space group symmetries of the framework will naturally determine the symmetrically equivalent positions $r\in R^{3}$ that consequently have the same energy. In this manner, the Ionic TuTraSt approach lends itself well to utilization of symmetry in the sampling of the potential energy surfaces since the single-particle potential necessarily must have the same symmetry as the host framework structure.

TuTraSt analyzes the potential energy surface sampled on a regular grid, rectilinear in fractional space, e.g., with regular spacing in the unit cell vector directions. Two grid points ***p*** and ***p*** * are symmetrically equivalent if there is an element $g:R^{3}\to R^{3}$ of the space group of the structure such that *g*(***p***) = ***p*** *. It is then sufficient to sample a single representative of each equivalence class of symmetrically equivalent grid points (see Figure 1).

**Figure 1:**
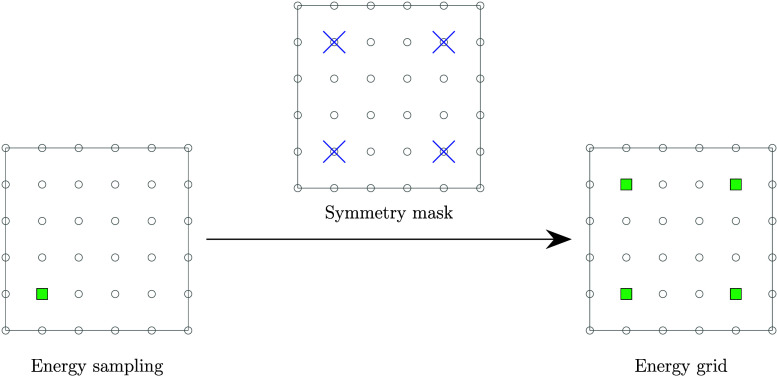
Schematic representation of the application of space group symmetry to energy grids. With the use of a symmetry mask, a single representative point can be sampled (green square on the left) from each set of symmetrically equivalent points (blue crosses), and the result can be assigned to each of the equivalent points.

### 2.2 Grid Exclusion

Strategies to exclude grid points from the explicit sampling of energy can be viewed from the perspective of high- and low-energy regions, where the goal is to exclude the much less relevant high-energy regions with negligible importance. As illustrated by the Boltzmann distribution, the weight and probability of a configuration decay exponentially with its energy, and hence, low-energy configurations dominate in occurrence and contribution to the properties of the system. It is therefore important to avoid unnecessary sampling of high-energy configurations, especially when the sampling is computationally expensive. When properties are sampled as a function of the position of a guest particle in a rigid structure, it is particularly useful to avoid sampling within a volume that is (energetically) blocked by the host structure.

A recent application of this principle in the context of grid sampling is in the GrAED algorithm by Ren and Coudert,^6^ in its application to calculate the Xe/Kr adsorption energies for metal–organic frameworks. High-energy points were identified by calculating the repulsion between the sampled guest atom and the closest framework atom, excluding grid points above a certain repulsion energy.

This approach works well for weakly interacting guest species, such as noble gas atoms, and when the interactions are modeled with an additive force field. When using DFT, this approach does not work, as it is difficult or even impossible to decompose the energy into distinct parts, such as a specific atom-pair repulsion. Moreover, for ion diffusion, the ion–ion interactions between the mobile ions strongly affect the energy landscape and the diffusion, as we have discussed previously.^11^

In order to use a similar strategy based on the energy, it would be necessary to approximate the multiparticle potential with an effective potential including the effect of the interactions between a stoichiometric number of mobile ions, which adds significant complexity and is unclear how to practically achieve. Instead, we employ a purely geometrical approach by excluding points within spherical cutoffs around the framework atoms based on scaled van der Waals radii of the framework atoms, which estimates the blocked high-energy region around them (see Figure 2). That is, the cutoff radius *r*_*c*_ around a framework atom is given by

1 $$r_{c}=s\cdot r_{vdW}$$

where *s* is the scaling factor and *r*_*vdW*_ the van der Waals radius of the atom. The van der Waals radii are used to estimate a cutoff for high energy interatomic distances between framework atoms of varying sizes and the mobile Li corresponding to highly improbable overlap regions, providing spheres around the framework atoms within which sampling is entirely avoided. We test the limitations of this approach, the sensitivity of the diffusion coefficients on the scaling factor *s*, and determine an approximate maximum permissible cutoff scaling factor.

**Figure 2:**
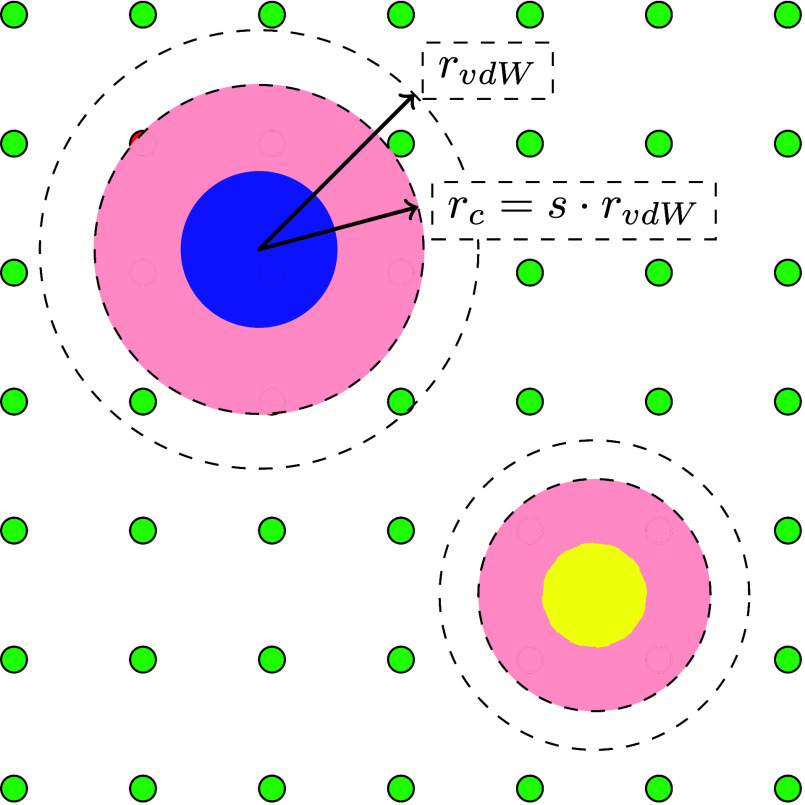
Schematic representation of the scaled vdW radius exclusion approach used in this work. The blue and yellow circles indicate the framework atoms. Their van der Waals radii are indicated, and the red shaded regions around them show the excluded regions in which sampling is not performed. These are given by a cutoff radius *r*_*c*_ = *s*·*r*_*vdW*_, where *s* is the scaling factor. Small green disks and small red disks represent sampled and unsampled grid points, respectively.

### 2.3 Interpolation

A regular grid structure of the potential energy data allows for a particularly straightforward application of interpolation. Ionic TuTraSt uses the precomputed single-particle potential energy grid as part of the potential in the MMC simulation, in which the Li-coordinates can vary continuously in the entire simulation cell, requiring some form of interpolation. A smooth interpolation of the single-particle potential furnishes a smooth interaction potential in this simulation. So far, a simple nearest-neighbor evaluation was used for this purpose, in which the single-particle potential at any coordinate was assigned the value of the closest grid point. Sampling at a sparser resolution reduces the number of grid points required for computation, lowering the computational cost of sampling the single-particle potential energy grid used as the input for Ionic TuTraSt. Applying a smooth interpolation ensures a smooth representation of the potential energy surface, mitigating the accuracy loss associated with coarser grid spacing. We choose cubic interpolation as it produces a smooth interpolation while being only slightly more computationally expensive than a linear or nearest-neighbor approximation. Using higher-order polynomial approximations is not only more costly and problematic to combine with exclusion of sample regions but also prone to overfitting and therefore not desirable in this setting.

In this work, we use the implementation of a 3-dimensional, smooth tricubic interpolation in RASPA,^19^ which in turn implements a scheme by Leiken and Marsden.^20^ Interpolation of the single-particle energy is performed during the Metropolis Monte Carlo correction step, which is carried out in RASPA. In order to evaluate the performance and limitations of this interpolation method, as well as the effect of grid spacing on the accuracy of the final diffusion coefficients, we sample single-particle grids at varying resolutions and compare the results obtained using tricubic interpolation with those obtained using the nearest-neighbor approach within the MMC-correction scheme.

## 3 Computational Details

We implement grid sampling reduction strategies, as discussed in the Theory section, in a workflow for constructing single-particle DFT grids for subsequent use in Ionic TuTraSt, which below will be referred to as DFT-grid Ionic TuTraSt. We tested the combined approach for the prediction of ion diffusion as follows.

First, to explore the speed-up, sensitivity, impact, and limitations of the grid reduction strategies, we perform DFT-grid Ionic TuTraSt on the structure set previously used to validate the Ionic TuTraSt procedure in our earlier work,^11^ consisting of 84 Li-containing inorganic crystalline structures from the Materials Cloud database.^21^ These were selected from Li-containing crystalline inorganic compounds containing in total three or four different elements and an even number of electrons in order to avoid the need for spin-polarized DFT calculations. From the previous study, this set was also deemed to represent a desirable variety of structures and compositions, comprising 56 different elements. For an array of combinations of grid spacing and exclusion parameters, the resulting diffusion coefficients are compared, and the impact of the parameters is evaluated.

Second, a set of 14 inorganic, crystalline, fast Li-ion conductors was constructed for validation of our computational approach to AIMD simulations. The selection, listed in Table 1, consists of structures of varying families identified in the literature to be superionic conductors, which entails in most cases that the ion diffusivity is fast enough for it to be probed within the time scale of an AIMD simulation, typically in the order of hundreds of picoseconds. In addition, we have included the two structures that showed the highest diffusivity with DFT-grid Ionic TuTraSt from the first structure set (Li_3_H_4_Rh and LiYF_2_).

**Table 1:** List of 14 Crystalline Inorganic Structures Identified in the Literature to Be Fast Li-Conductors and which Are Used to Validate Our Ionic TuTraSt Method and Grid Sampling Reduction Tools toward ab Initio Molecular Dynamics

Structure	Reference
Li_1.3_Al_0.3_Ti_1.7_(PO_4_)_3_	He et al.^22^
Li_10_GeP_2_S_12_	Weber et al.^23^
*cubic*-Li_7_La_3_Zr_2_O_12_	Burbano et al.^24^
Li_12_Si_7_	Kirsch et al.^25^
Li_13_Si_4_	Kirsch et al.^25^
Li_2_HN	Chen et al.^26^
Li_3_La_5_Cl_18_	Yin et al.^27^
Li_6_PS_5_Cl	Baktash et al.^28^
Li_7_P_3_S_11_	Zhou et al.^29^
LiFe(CN)_3_	Zhang et al.^30^
LiMnO_2_	Zhang et al.^30^
LiNbO_3_	Zhang et al.^30^
Li_3_H_4_Rh	this work
LiYF_2_	this work

We apply DFT-grid Ionic TuTraSt to this set of structures and compare isosurfaces and Li-ion diffusion coefficients with those obtained from the ab initio molecular dynamics (AIMD) simulations, in order to validate the general approach. The following sections describe the computational details of each step of the procedure.

### 3.1 Grid Sampling

The calculations of single-particle potential energy grids are conducted as follows. First, the structure is transformed to the corresponding conventional unit cell representation (as defined by Setyawan et al.^31^) using the Python Materials Genomics (pymatgen) python library.^32^ We utilize the spglib python package^33^ for space group determination, while the determination of symmetrically equivalent grid points, handling of symmetry masks and grids are implemented using the GEMMI project python library.^34^ Based on the chosen grid spacing, the number of grid points in each unit cell vector direction is determined as the smallest number of points that yield a spacing less than or equal to the chosen spacing and simultaneously is compatible with the space group symmetries. Here, compatibility means invariance of the grid under the action of the space group, i.e., every symmetry operation maps all grid points to grid points. After determining the space group as well as the number of grid points in each direction, we identified the grid points to be excluded from sampling based on the radial cutoff. Using this information, we then construct a minimal set of symmetrically independent grid points for sampling, as described in the following.

For the radial exclusion of grid points, a mask is generated by looping over all framework atoms and finding the grid points within the set cutoff distance of the framework atom, taking into account periodicity. In the case of the scaled van der Waals exclusion, the cutoff distance is *s* × *r*_*vdW*_ where *s* is the chosen scaling factor and *r*_*vdW*_ is the van der Waals radius of the framework atom, as defined in the mendeleev python package.^35^ The mask constructed in this manner contains information about the grid points that are not to be sampled due to being inside the excluded volume. Symmetrically unique points are identified by looping through all grid points and using a symmetry mask in the following manner; A point is skipped if it has already been marked as sampled in the symmetry mask or has been flagged to be excluded in the previously created exclusion mask. Otherwise, it is added to the list of points to be sampled, and all symmetrically equivalent entries are marked as sampled in the symmetry mask. Before sampling, all Li-atoms in the structure are removed to obtain the framework structure. Each grid point is sampled by placing a Li-atom at the corresponding coordinates in the framework structure, and then the energy of the resulting configuration is calculated. The framework structure is kept fixed. A geometry optimization of the single particle systems would lead to a biased and unphysical multiparticle grid as each ion would have a different energy minimized environment, while also ignoring the effect of simultaneous presence of all ions on the framework. After sampling, the minimum energy among the sampled points is shifted to zero. The symmetry mask is then used to construct the grid by assigning each sampled energy to all grid points in the same symmetry equivalence class as the sampled point. The remaining grid points, belonging to symmetry equivalence classes that are not sampled, are assigned a very large positive value.

To compute the single potential energies for the selected grid point configurations for each structure, density functional theory calculations are performed utilizing the CP2K software package^36^ (version 2023.1). The Perdew–Burke–Ernzerhof (PBE) functional,^37^ with Grimme’s D3 dispersion correction,^38,39^ is used, and the plane-wave cutoff is set to 400 Ry and the relative cutoff to 50 Ry in all calculations. For La and Lu, lanthanide basis sets DZV-MOLOPT-GTH and DZV-MOLOPT-SR-GTH, respectively, are used,^40^ while for all other atoms the corresponding DZVP-MOLOPT-SR-GTH basis sets are used.^41^ Goedecker–Teter–Hutter pseudopotentials optimized for PBE (GTH-PBE)^42–44^ are used for all atoms. Convergence tests presented in the SI show stability toward 800 Ry plane-wave cutoff (Figure S18), the SCAN functional (Figure S19), and larger cell sizes (Figure S20). To compensate the net negative framework charge when only including a single Li-ion in the sample, a background charge is added. The charge of the grid sampling configuration, consisting of the framework structure with a single Li-ion, was set to *q*_*BG*_^*full*^ = 1 – *n*_*Li*_ where *n*_*Li*_ is the stoichiometric number of Li in the simulation cell. This we refer to as a *full* background model, i.e., assuming that each stoichiometric Li is a fully ionized monovalent cation and gives one full electron to the framework.

For the set of 14 fast Li-ion conductors we also compute single-particle grids where the background charge is scaled by the averaged Li-atom partial charge of each structure, computed by the REPEAT method^45^ *q*_*Li*_^*repeat*^, according to $q_{\mathrm{BG}}^{\mathrm{repeat}}=\lfloor \left(1-n_{\mathrm{Li}}\right)\times q_{\mathrm{Li}}^{\mathrm{repeat}}\rceil$ . This ionization model is termed the *repeat* background model.

When scaling *q*_*BG*_^*full*^ by the averaged Li REPEAT charge, rounded to the nearest integer, several of the resulting systems contained an odd number of electrons. Thus, these systems required spin-polarized calculations, resulting in calculations much slower than those where only monovalent cations are removed. To investigate if the ionization effects of the repeat background model could be approximated to a lower computational cost, we define a third background model by scaling the PES computed using the full background model by the value of the average Li REPEAT charge. This ionization model we refer to as the *scaled* background model.

### 3.2 Ionic TuTraSt and Interpolation

The sampled single-particle DFT grids are subjected to the Ionic TuTraSt workflow:^11^ a Metropolis Monte Carlo (MMC) simulation is used to sample the ion–ion interactions of the stoichiometric number (*n*_*Li*_) of Li-ions within the single-particle PES, producing a corrected multiparticle energy grid. This corrected multiparticle energy grid is consequently analyzed by a topological analysis (TuTraSt) that allows one to obtain transition rates between Li sites, and based on this information, a kMC simulation is performed to compute the Li self-diffusion coefficient. The MMC simulations were carried out in the NVT ensemble at 1000 K to ensure sufficient sampling of the PES using the RASPA software.^19^ The MMC moves are performed on *n*_*Li*_ Li in an empty supercell with all other atoms removed. The supercells are constructed to fulfill the minimum image convention using a Lennard-Jones cutoff of 12.5 Å, and *n*_*Li*_ is adapted accordingly to maintain stoichiometry. The Li–Li interactions were modeled with electrostatic interactions computed with Ewald summation, assigning the same constant partial charge to each *Li*, and a Lennard-Jones potential with parameters from the Universal Force Field^18^ in order to include short and long-range van der Waals interactions. For the test set of 84 structures, the Li-partial charge was set to *q*_*Li*_ = 0.5*e* as this was shown in our previous study to be the most stable constant value.^11^ For the set of the 14 fast ion conductors we used partial charges *q*_*Li*_ = 0.0, 0.5, 1.0*e* and partial Li charges *q*_*Li*_ = *R*. Diffusion coefficients were calculated as the average of those obtained from 10 independent kinetic Monte Carlo simulations. This procedure, as well as the computation of the corrected multiparticle energy grids from the resulting MMC trajectories and the following TuTraSt analysis of these grids, follows that in our previous work.^4,11^

Interpolation of the single-particle grids was implemented as an integrated step into the MMC simulation procedure performed with RASPA. The routine in RASPA to handle grids was previously modified to enable reading of external potential grids.^11^

We also modified the routine in RASPA that calculates potential energy derivatives and uses them in tricubic Lekien–-Marsden interpolation.^20^ The modification allows RASPA to read in precomputed grids of potential energy derivatives, i.e., grids containing the values of the derivatives at the grid points. These derivative grids were generated using finite difference methods, as described below.

The sampled single-particle DFT grid was first read and converted to the orthogonal grid format used in RASPA. The details of this format and the procedure used to convert grids in nonorthogonal unit cells are described in the SI. In the following, the resulting orthogonal energy grid is denoted {*E*_(*i*,*j*,*k*)_: (*i*, *j*, *k*) ∈{0, ..., *N*_*x*_ – 1} × {0, ..., *N*_*y*_ – 1} × {0, ..., *N*_*z*_ – 1}}, where *E*_(*i*,*j*,*k*)_ is the energy at the grid point with coordinates ***p***_(*i*,*j*,*k*)_ = (*id*_*x*_, *jd*_*y*_, *kd*_*z*_), where *d*_*x*_, *d*_*y*_, and *d*_*z*_ are the spacings between adjacent grid points in each coordinate direction, and *N*_*x*_, *N*_*y*_, and *N*_*z*_ are the total number of grid points in the respective directions. The grids of first order partial derivatives were then computed using a central difference approximation, given in eq 2 for the *x*-derivative.

2 $${\left(\frac{\partial E}{\partial x}\right)}_{\left(i,j,k\right)}\approx \frac{E_{\left(i+1,j,k\right)}-E_{\left(i-1,j,k\right)}}{2d_{x}}$$

Here, *d*_*x*_ is the grid spacing in the *x*-direction. The first derivatives with respect to *y* and *z* were computed analogously, by changing the instances of *x* with *y* or *z*, and instead taking the difference with respect to the *j*- or *k*-index, respectively.

Similarly, the grid of mixed second-order derivatives in *x* and *y* were calculated using eq 3. The corresponding mixed derivative grids in *xz* and *yz* were calculated analogously.

3 $${\left(\frac{\partial^{2}E}{\partial x\partial y}\right)}_{\left(i,j,k\right)}\approx \frac{E_{\left(i+1,j+1,k\right)}-E_{\left(i+1,j-1,k\right)}-E_{\left(i-1,j+1,k\right)}+E_{\left(i-1,j-1,k\right)}}{4d_{x}d_{y}}$$

Lastly, the third-order mixed derivative grid was computed according to eq 4.

4 $${\left(\frac{\partial^{3}E}{\partial x\partial y\partial z}\right)}_{\left(i,j,k\right)}\approx \frac{1}{2d_{z}}\left({\left(\frac{\partial^{2}E}{\partial x\partial y}\right)}_{\left(i,j,k+1\right)}-{\left(\frac{\partial^{2}E}{\partial x\partial y}\right)}_{\left(i,j,k-1\right)}\right)$$

The potential energy grid and the grids containing the derivatives were then used in the existing implementation of Leiken–Marsden-type tricubic interpolation within RASPA for the evaluation of the single-particle potential in the MMC simulation. Special care was taken in the proximity of excluded (i.e., unsampled) grid points to avoid numerical issues and the propagation of errors: The tricubic interpolation was used when the single-particle potential was evaluated within voxels, for which derivatives at all corners could be calculated from sampled points. In cases where one or more of the points required in the calculations of the derivatives were not sampled, nearest-neighbor evaluation was employed instead within the voxel. This procedure is illustrated in Figure 3.

**Figure 3:**
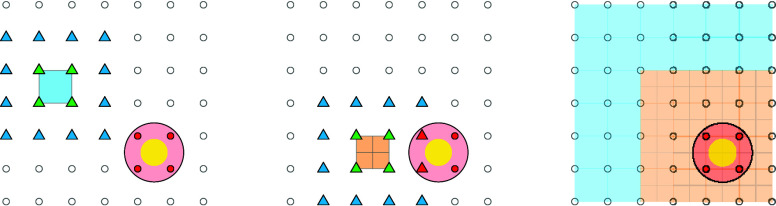
Schematic figure showing the interpolation procedure for points outside the grid in combination with the exclusion of sampled grid points (red). The yellow disk indicates a framework ion, and the red shaded circle is the exclusion region around it. Note that the picture only is intended to show a portion of the grid and as such does not depict periodic boundary conditions. Left: Tricubic interpolation is used inside the blue-shaded voxel since all grid points (blue and green triangles) that are needed for calculating the derivatives at the corners of the voxel (green triangles) are sampled. Middle: Nearest-neighbor interpolation is used inside the orange-shaded voxel since two of the grid points required to compute the derivatives on the corners of the voxels are not sampled (red triangles). Right: All voxels colored based on whether tricubic interpolation (blue) or nearest-neighbor interpolation (orange) is employed within them.

### 3.3 Ab Initio Molecular Dynamics

AIMD simulations were performed with the same settings as the single-point calculations in the grid sampling. Supercells were used for structures with unit cell distances >7–8 Å to avoid self-interaction effects on the Li dynamics. The exception is Li_3_H_4_Rh, where the high computational cost for the supercell of this structure limited the simulation time to 50 ps, which is too short to observe diffusion. The AIMD simulation of this structure is thus run on the unit cell despite the short cell distances, and the resulting diffusion coefficient may thus not be reliable. The unit cell parameters and AIMD supercells for each structure are provided in Table S2. Each simulation cell contained the respective stoichiometric amounts of Li ions *n*_*Li*_. For direct comparison with Ionic TuTraSt, all atoms, except for the Li atoms, were fixed. The simulations were conducted at a temperature of *T* = 1000 K with a time-step of *dt* = 2 fs. All systems were first equilibrated for 20 ps in an *NVE* ensemble, rescaling the temperature when it is more than 10 K above or below the target temperature. After equilibration, production simulations were run in an *NVT* ensemble with a CSVR thermostat for a simulation time of *t* = 100–1000 ps depending on the time needed to reach the diffusive regime in at least one direction as defined in Table S1.

The Li trajectories were printed every 100 fs in order to calculate the mean squared displacement *MSD*(τ) of the Li-atoms. *MSD*(τ) is the average mean square displacement over all pairs of frames from the printed trajectory that are separated by time τ. The self-diffusion coefficient (*D*_*s*_) is then computed from the slope *MSD* = 6 × *D* × τ, within a fitting window of this function where the diffusive regime has been reached, i.e., linear scaling. The MSD of a typical diffusion process first extends through several regimes which on a prediffusive time scale can appear linear. These so-called ballistic regimes correspond to jumps between energy minima but without diffusion paths that percolate through the full unit cell. Plotting *MSD*(τ) on the log–log scale is thus often crucial to validate that the fitting window is, in fact, part of the linear regime, i.e., where the log–log slope equals unity.

For additional validation of the DFT-grid Ionic TuTraSt output, three-dimensional PES grids are generated from the AIMD trajectory data. To improve the statistics, the symmetry mask is applied when computing the probability, where the Li count of visiting each grid volume is averaged over all equivalent symmetries. The same procedure is carried out when constructing the multiparticle PES grid from the MMC output in the Ionic TuTraSt procedure as described in detail by Gustafsson et al.^11^

## 4 Results and Discussion

### 4.1 Performance and Limit Tests of Grid Sampling Strategies

#### 4.1.1 Symmetry

For the reduction tool validation set of 84 structures, the distribution of the number of grid point calculations required for a grid resolution of 0.2 Å, with and without applying symmetry reduction, is presented in Figure 4. The symmetry reduction factor for the individual structures ranges from 2 to 150 and averaging at 26.

**Figure 4:**
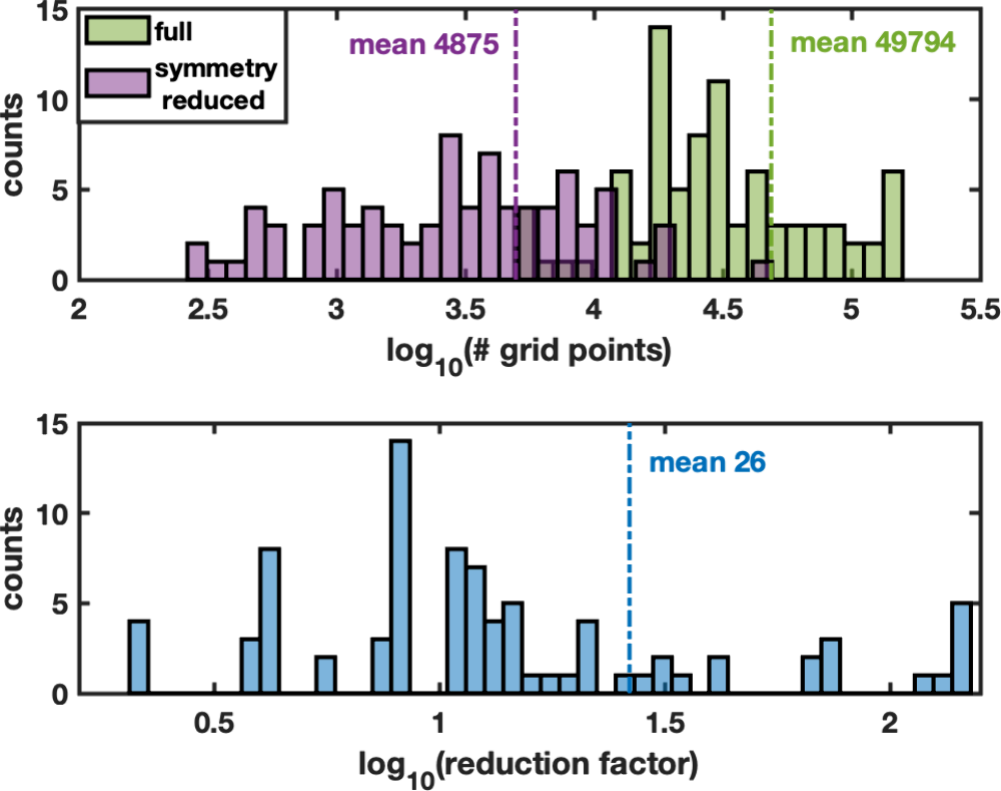
Savings using symmetry for the single-particle grid sampling of the test set of 84 structures. Top: Distribution of the total number of grid points and number of grid points after symmetry reduction for 0.2 Å grids. Bottom: Distribution of the reduction factor due to symmetry.

#### 4.1.2 Grid Exclusion

The effects of this exclusion strategy on the resulting diffusion coefficients using different scaling factors at different grid resolutions are shown in Figure 5.

**Figure 5:**
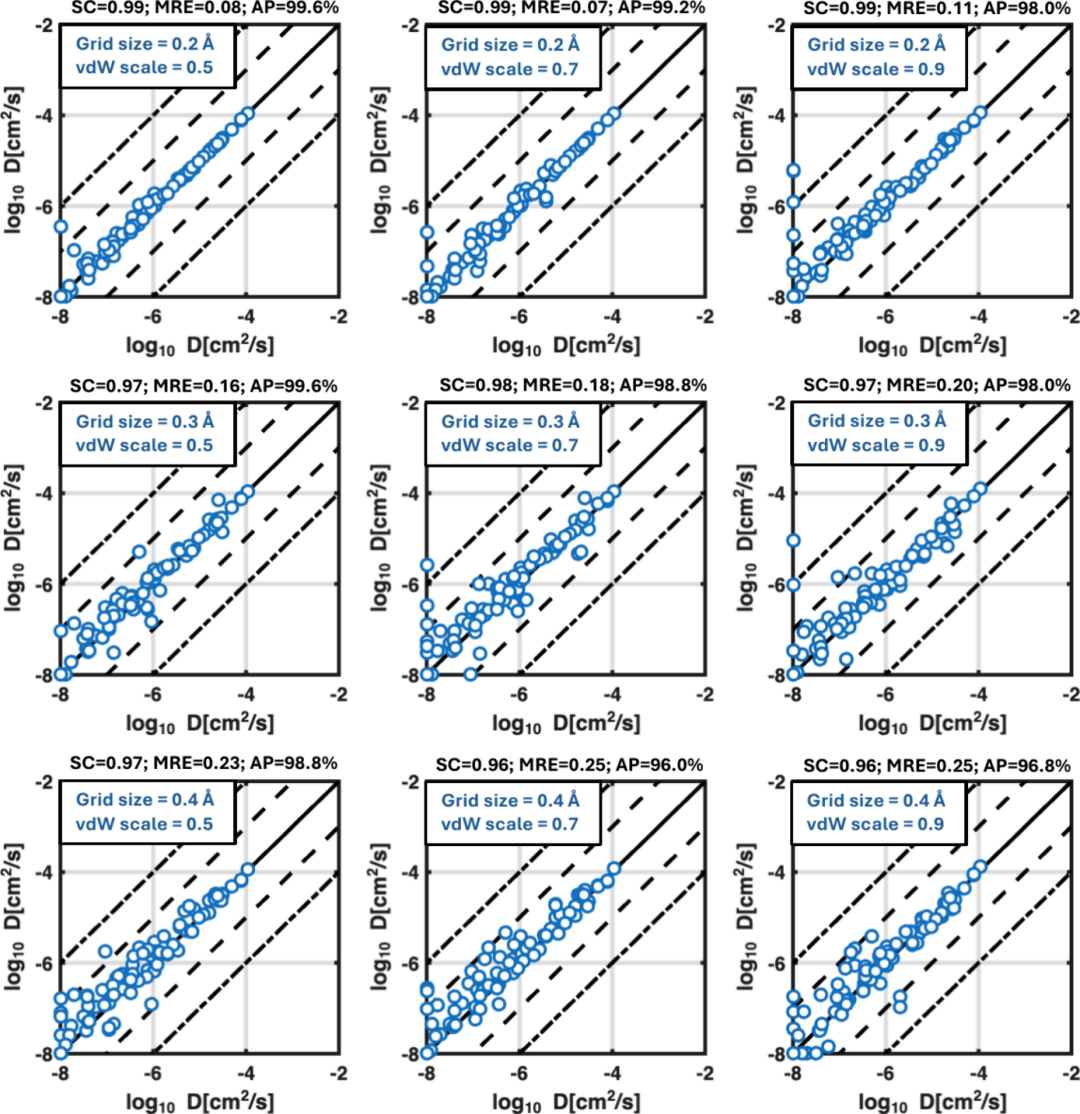
Diffusion coefficients computed by DFT-grid Ionic TuTraSt applied to different vdW cutoffs and grid resolutions plotted against the results for the finest resolution (0.2 Å) and without vdW exclusion. Results are shown for a combination of grid resolutions of 0.3 Å, 0.4 Å, and 0.5 Å (top to bottom) and scaling factors of 0.5, 0.7, and 0.9 (left to right).

The diffusion predictions for 0.2–0.3 Å resolution combined with vdW scalings of 0.5–0.7 for the cutoff appears to not change much compared to the reference and remains stable for the vast majority of the structures. When increasing the vdW scaling factor to 0.9 for 0.2 Å and 0.3 Å grid resolutions, the error in the predictions at low diffusion (for *D*_*s*_ < 10^–6^ cm^2^/s) increases with several deviations of more than 1 order of magnitude. At higher diffusion, the results for the remaining predictions still show a good correlation with those of the reference. Going to 0.4 Å grid resolution, the results become poorer, and there are also some false negative predictions, indicating that this cutoff scaling is beyond or close to the applicable limit, considering accuracy. Note that as the grid spacing becomes larger, the effect of excluding a grid point will increase not only because the corresponding voxel represents a larger volume but also because the region estimated using the more accurate tricubic interpolation scheme becomes smaller while the region using the nearest neighbor approximation becomes larger. The 0.4 Å grid spacing with reduced vdW scalings of 0.3–0.5 retains a good match with the reference again. While the agreement decreases for 0.7 vdW scaling, the results stay within a 1 order of magnitude error margin. This suggests that using a 0.4 Å spacing with a vdW cutoff scaling of 0.7 provides good stability relative to the finer 0.2 Å grid without vdW exclusions, indicating a good balance between accuracy and efficiency for screening studies.

The corresponding reduction of grid points, as a function of the cutoff scaling factor, is given in Figure 6. For a scaling of 0.5, on average 17% of the grid points are excluded, while for 0.7 almost half are excluded.

**Figure 6:**
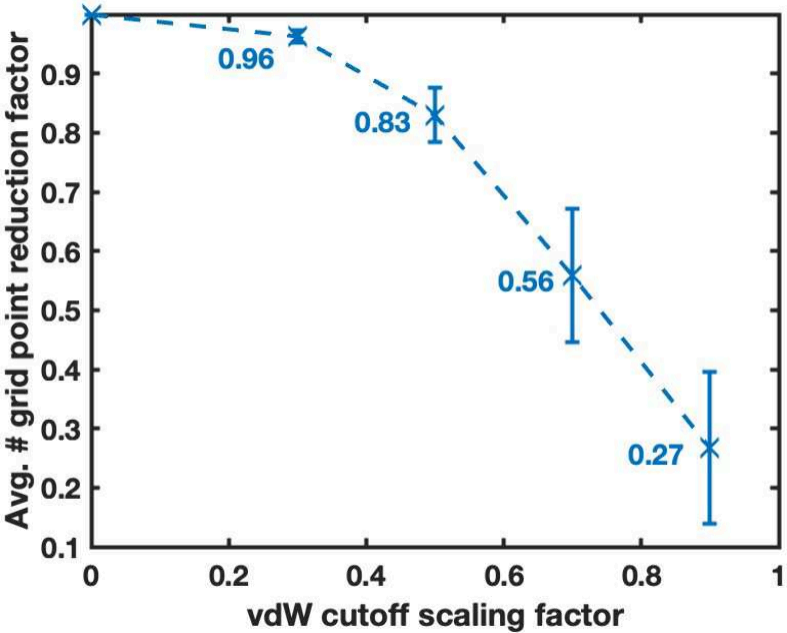
Average inverse reduction factor (reduced number of points/total number of points) vs cutoff scaling factor, for the vdW exclusion applied to the test set. Bars indicate one standard deviation.

#### 4.1.3 Interpolation

To test the effect of applying interpolation schemes during the MMC sampling, we compare the diffusion coefficient output of Ionic TuTraSt using multiparticle grids computed with the tricubic interpolation described in Section 3.2 with those computed using a nearest neighbor evaluation only, e.g., every point in a voxel is simply assigned the constant energy value of the nearest grid poin. Since the stability of the interpolation is expected to depend on grid size, we compare the results of using interpolation at grid resolutions of 0.3 Å, 0.4 Å, and 0.5 Å to those obtained with the finest resolution, 0.2 Å, which serves as our validation data.

To evaluate the stability of the interpolation, we define three quantities: (1) The Spearman coefficient (SP) which quantifies the correlation of ordering, (2) mean relative error (MRE) which is the mean absolute relative deviation between the data points, and (3) accurate predictions (AP) the percentage of data points that lie within 1 order of magnitude from corresponding validation points.

From Figure 8 it can be seen that the tricubic interpolation significantly improves and stabilizes the results as the grid spacing is increased up to 0.4 Å: Increasing the grid size to 0.3–0.4 Å shows only minor losses in precision compared to 0.2 Å, and practically all (∼99%) diffusion coefficients lie well within a 1 order of magnitude margin from the reference, and show good correlations with *SC* = 0.98 and 0.96, and small mean relative errors of *MRE* = 0.13 and 0.23, respectively. This indicates that 0.4 Å is a feasible grid spacing when employing the tricubic interpolation scheme.

When using only nearest neighbor interpolation, the results quickly worsen with increased grid spacing. They become noisier and more unstable, and all stability measures decline. While the results for 0.3 Å are maintained within 1 order of magnitude, 0.4 Å shows several large outliers and even several false negatives. When going further to 0.5 Å grid resolution, the beneficial effects of tricubic interpolation seemingly fade, and both the nearest neighbor and tricubic interpolation accuracies decrease significantly. This is not surprising, considering that this is a rather coarse spacing compared to the length scales of the atomistic interactions, and the tricubic interpolation cannot improve this if the sampling is too sparse. In fact, such a sparse sampling might even amplify errors, explaining why the stability quantities are slightly better with nearest neighbor interpolation than with tricubic interpolation at a 0.5 Å grid spacing.

These results indicate that the employed tricubic interpolation scheme, together with DFT-grid Ionic TuTraSt, allows the grid spacing to be relaxed to 0.4 Å with only minor errors. This reduces the number of calculations by a factor of 8 when compared with a resolution of 0.2 Å. It is also conceivable that a 0.5 Å spacing, allowing for a reduction factor of 16, could be used in a coarse, approximate prescreening to screen for only the fastest diffusers, given that for *D*_*s*_ > 10^–6^ *cm* ^2^/*s* the accuracy is still within 1 order of magnitude for both interpolation strategies. Combining all three strategies, the total reduction achieved is shown in Figure 7, using the found optimized settings of 0.4 Å grid spacing and 0.7 cutoff vdW scaling.

**Figure 7:**
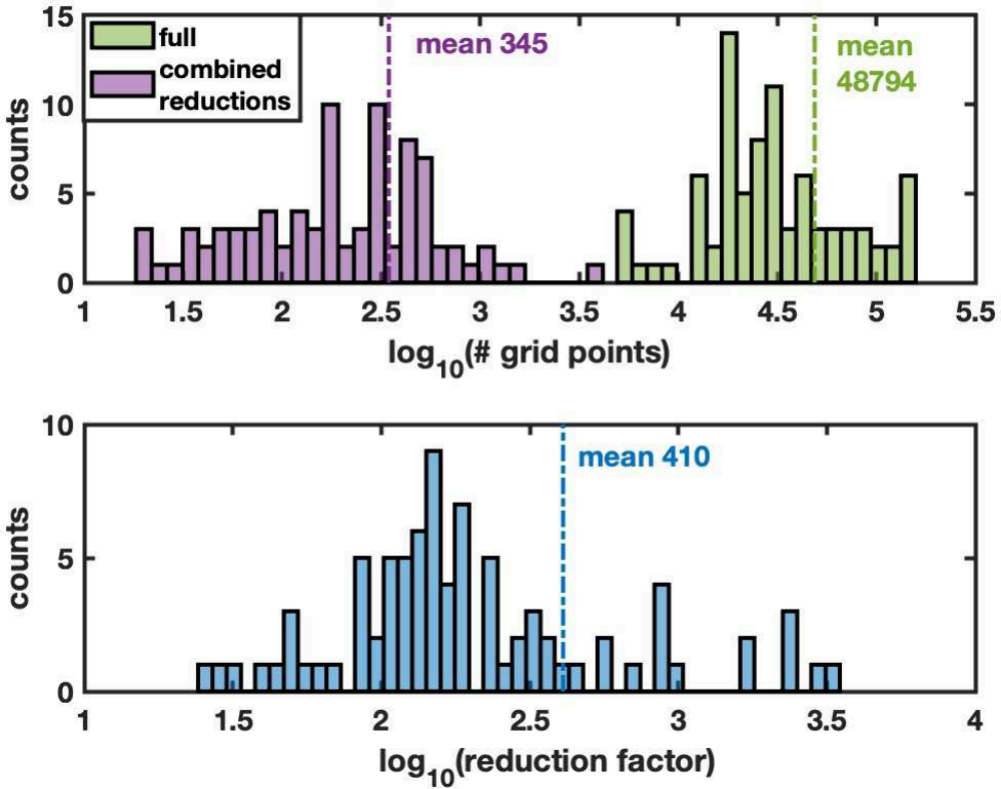
Top: The distribution of sampled grid points for the test data set of structures, comparing sampling at 0.4 Å, with 0.7 van der Waals scaling and using interpolation and symmetry, with sampling at 0.2 Å grid spacing without any reduction. Bottom: Distributions of reduction factors for the test data set of structures, for grid sampling at the found parameters 0.4 Å with 0.7 van der Waals scaling and using interpolation and symmetry, compared to a 0.2 Å grid without any reduction employed.

**Figure 8:**
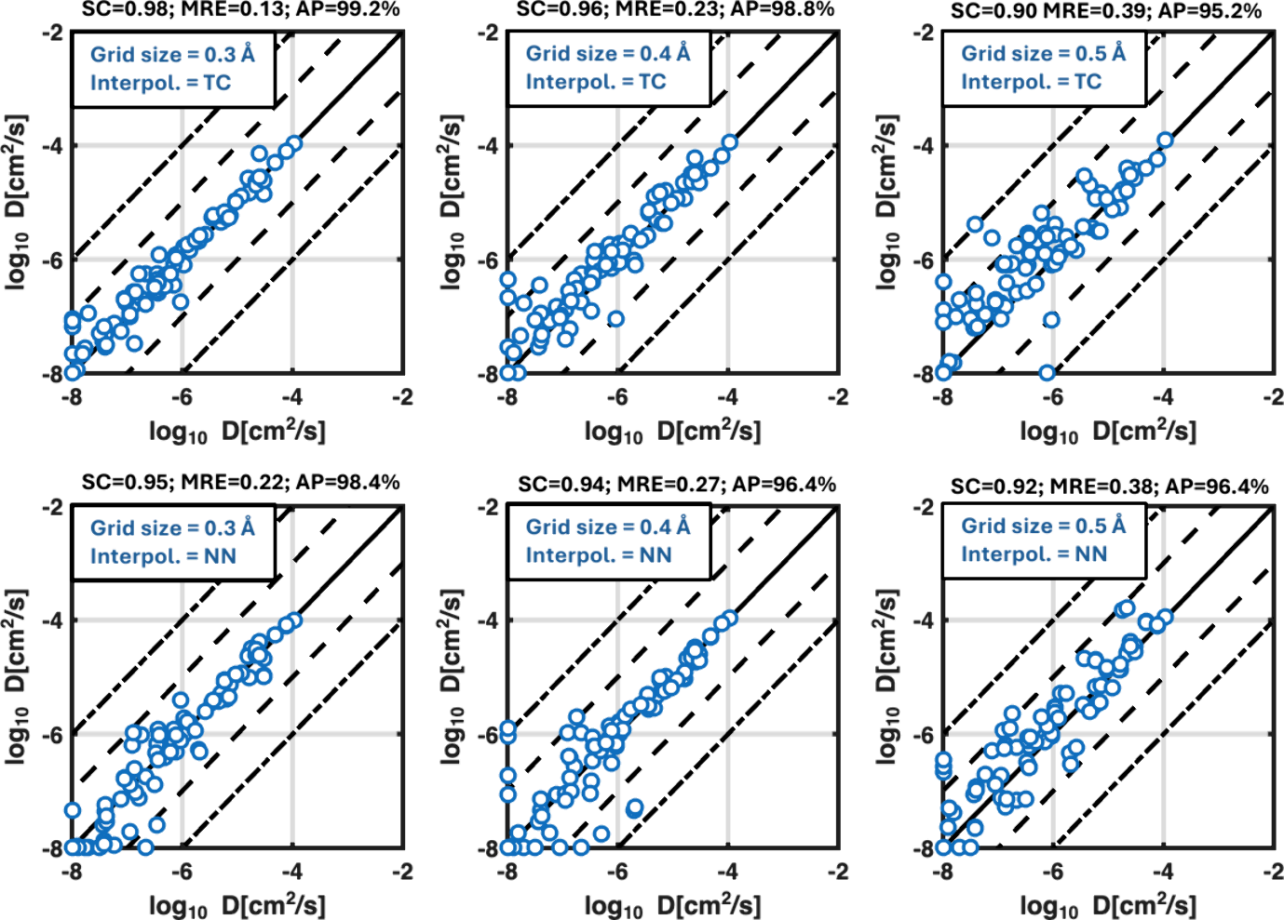
Effect of the grid spacing and interpolation. Predicted diffusion coefficients from Ionic TuTraSt applied to DFT single-particle potential grids of different resolutions, plotted against the results for the finest resolution (0.2 Å). The plots on the top row show results using tricubic interpolation (TC). Plots on the bottom row show results corresponding to a nearest neighbor (NN) evaluation.

### 4.2 Comparison with AIMD in Cases of Fast Ion Diffusion

To validate the accuracy and computational efficiency of DFT-grid Ionic TuTraST we compare Li diffusion computed using the optimized grid reduction settings, i.e., vdW cutoff scaling factor 0.7 and grid resolution 0.4 Å, with corresponding AIMD diffusion data.

#### 4.2.1 Computing Diffusion Coefficients from AIMD Trajectories

Obtaining convergence of self-diffusion coefficients (*D*_*s*_) from AIMD trajectories is often challenging, as the computational cost limits the simulation length and, in turn, the statistics of rate-determining process occurrences. This also limits the possibility of applying robust and uniform convergence criteria, such as we have done in our previous work where the fitting window is defined as the part of the mean square displacements (*MSD*) corresponding to the particles, on average, diffusing one to two times the unit cell parameter in each direction.^4,11^

As none of the AIMD *MSD*s fulfill this strict criteria, we carefully analyze the trajectory output when choosing an appropriate fitting window for each structure. In addition to checking the linearity of *MSD*(τ) on both the linear and log–log scales, we estimate the minimal diffusion unit distance (this may be smaller than the unit cell parameter due to symmetry) and ensure that the mean absolute displacement within the fitting window is larger than this distance. Also, including *MSD* points toward the end of the τ interval is avoided as the number of data points used for averaging decrease. The precise interval that needs to be discarded depends on the number of Li-ions in the system as well as the diffusion rate and thus differs from system to system. The *MSD* plots on the linear and log–log scales including the fitting windows used to compute the diffusion coefficient for each structure, are presented in Figures S4–S17. One of the studied structures in the validation set Li_2_HN showed no diffusion during the AIMD simulation time of 400 ps.

As the diffusive regime of the directional diffusion is not reached in all three directions for most of the systems, we instead compute and compare the total three-dimensional *D*_*s*_ values with the corresponding values computed with DFT-grid Ionic TuTraSt. Although the latter diffusion model is successful in including all diffusion processes, even those with activation energies too high to be probed with AIMD, we motivate that this total *D*_*s*_ is the most robust comparison metric at hand as it will be dominated by the fastest diffusion, i.e., those that actually have reached convergence within the AIMD simulation time scale.

Another challenge with the AIMD computed diffusion coefficients is the difficulties in estimating the statistical errors, as this would require multiple parallel simulations multiplying the already demanding computational cost by the equivalent factor. However, in cases where the structure is isotropic in several dimensions, the directional diffusion coefficients should also be equal, and the spread of these can thus give an estimation of the error. Of the 14 structures that we studied, three of them are isotropic in all three dimensions. The standard deviation of the directional diffusion coefficients then gives an approximate indication of the general size range of the AIMD errors as shown by the horizontal error bars in Figure 9.

**Figure 9:**
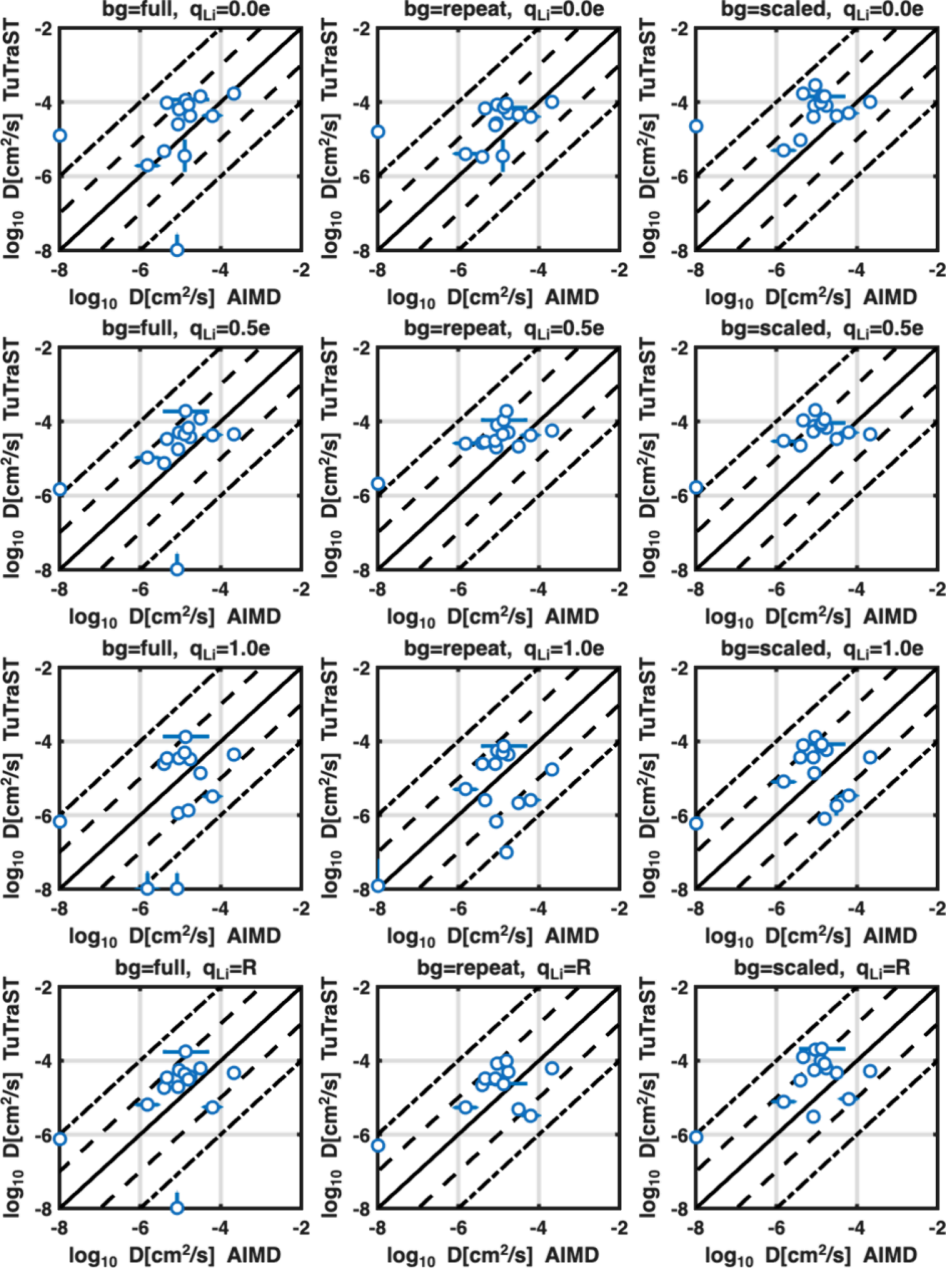
Log–log plots of three-dimensional diffusion coefficients from DFT-grid Ionic TuTraSt plotted against corresponding diffusion coefficients from AIMD for different partial charges (*q*_*Li*_ = 0.0*e*, 0.5*e*, and 1.0*e* and structure specific repeat charges *q*_*Li*_ = R) and background ionization models (bg = full, repeat, and scaled). Points on the plot boundaries (*x* = −8 and *y* = −8) correspond to *D*_*s*_ = 0 values on the respective axis.

#### 4.2.2 Effect of Li-Ion Partial Charge

As indicated by our previous work,^11^ defining the partial charge of the Li for the MMC sampling is not straightforward and where a fixed value of *q*_*Li*_ = 0.5*e* for all structures showed to give a stable result for prediction relative DFT-based Pinball MD.^46^ To get further insight into how to optimize the choice of *q*_*Li*_ with both accuracy and efficiency in mind, we extend that study for this data set. In addition, we investigate how the background charge used for the DFT-grid calculations, i.e., the *full*, *repeat* and *scaled* ionization models described in Section 3.1.

Starting with the full background model, similar to our previous study we vary a fixed partial charge from *q*_*Li*_ = 0.0–1.0*e* (*q*_*Li*_ = 0.0, 0.5 and 1.0*e* are shown in the left column of plots in Figure 9) as well as using DFT-based REPEAT charges *q*_*Li*_ = *R* computed individually for each structure. With *q*_*Li*_ = 0.0*e*, i.e., where electrostatic Li–Li are excluded and only vdW interactions are included with the Lennard-Jones model, it is surprising that the correlation with the AIMD, in fact, is fairly stable. Of the 14 structures studied, 11 are within the target agreement of our method with ab initio MD of 1 order of magnitude in the predicted diffusion coefficients, although skewed toward the upper limit of the target range. One prediction is only slightly above this limit, and considering the error indication from the directional diffusion coefficients of the isotropic structures, it is likely that this slight deviation lands within the statistical error margin. For the second outlier—Li_6_PS_5_Cl, a 3-dimensional isotropic structure, our model predicts >2 orders of magnitude lower diffusion compared to AIMD. This false negative prediction remains independent of which *q*_*Li*_ is used and is discussed further later on. The third outlier is a false positive prediction of Li_2_HN showing high diffusion (*D*_*s*_ > 1 × 10^–5^ cm^2^/s) although no diffusion is observed in the AIMD for *q*_*Li*_ = 0*e*.

However, when increasing the value of *q*_*Li*_, the DFT grid Ionic TuTraSt computed *D*_*s*_ value for Li_2_HN decreases to *D*_*s*_ ∼ 1 × 10^–6^ cm^2^/s for *q*_*Li*_ = 0.5*e*, which is at the limit of what can be computed by AIMD and can thus be considered within the tolerance range limit while stability is maintained for the remaining structures. At *q*_*Li*_ = 1*e*, on the other hand, there is an increase in false negative predictions, including Li_3_La_5_Cl_18_, LiFe­(CN)_3_ and LiYF_2_, where the prediction has a significant loss of stability. When structure-specific partial charges (*q*_*Li*_ = *R*) are used instead of fixed values, a stable prediction is maintained and spread throughout the target range.

An interesting observation from these results is that *D*_*s*_ for the majority of structures remains relatively stable and only the above-mentioned 4 structures: Li_2_HN, Li_3_La_5_Cl_18_, LiFe­(CN)_3_, and LiYF_2_, differ more than 1 order of magnitude between *q*_*Li*_ = 0.0*e* and *q*_*Li*_ = 1.0*e*, and thus are highly sensitive to choice of partial charge. While *q*_*Li*_ = 0.5*e* and *q*_*Li*_ = *R* show the best correlations it is somewhat counterintuitive that *q*_*Li*_ = 0.0*e* also gives stable predictions for the majority of structures which may indicate that the high ion concentration results in a homogeneous “sea” of long-range electrostatic interactions and where short-range repulsive vdW interactions become more important. Here, an overestimation of partial charges might contribute to the ions being locked in their respective minimum energy sites, while an underestimation neglects this effect in structures with particularly high Li concentrations such as the case for Li_2_HN. These results thus indicate that an acceptable approximation of the multiparticle PES can be done for most structures even without including the ion–ion electrostatics at all in the MMC step of the Ionic TutraSt procedure when using a DFT-based single-particle grid as input.

#### 4.2.3 Background Charge Ionization Models

To investigate whether the Li ionization treatment that we apply can be improved, the single particle grids constructed with the *repeat* background model were used as input for the MMC simulation together with the same partial charge sets as for the *full* background model. For that model also, *q*_*Li*_ = 0.5*e* and *q*_*Li*_ = *R* perform the best. In addition, by relaxing the background charge of the Li-to-framework interaction to the Li-ionization predicted by REPEAT, the diffusion prediction for the significantly outlying false negative prediction of Li_6_PS_5_Cl is corrected. To further understand why the *repeat* background model is successful in reproducing the high Li diffusion shown by AIMD of Li_6_PS_5_Cl, while the *full* background model does not, we analyze and compare the PES of the different models and with the different partial charge sets presented in Figure S22. The PES constructed from the output of the AIMD trajectory shows two types of diffusion with different energy barriers: a local diffusion within four distinct clusters (*E*_*a*_ = 17 kJ/mol) and the rate determining diffusion between these clusters (*E*_*a*_ = 49 kJ/mol). Compared with the full background model PES, the energy barriers for diffusion between the clusters are significantly higher, independent of *q*_*Li*_ (*E*_*a*_ ∼ 80 kJ/mol). The repeat background model, on the other hand, softens the background ionization, resulting in better reproduction of the AIMD PES and a close correlation of the respective *D*_*s*_ values.

The *repeat* background model shows a similar trend for the partial charges as for the *full* background model. *q*_*Li*_ = 0.5*e* and *q*_*Li*_ = *R* result in all diffusion coefficients being within the AIMD accuracy goal. *q*_*Li*_ = 0.0*e* gives stable predictions of most structures but again overpredicts the diffusion of Li_2_HN and when increasing the value of *q*_*Li*_, the instability and spread are increased toward lower values and at *q*_*Li*_ = 1.0*e* several false negatives appear.

A validation set of only 14 structures (limited by the high computational cost of AIMD) is too small to draw extensive conclusions on the accuracy of these different ionization/partial charge protocols to be used for high-throughput screening for prediction of a large number of potential ceramic superionic conductors. However, the results do indicate that the *repeat* background model adapted to the DFT-defined Li-charge where a partial charge lower than 1 can be interpreted as the corresponding reduction of the effective Li ionization and therefore may be a more physical model than the *full* background model, where the latter entails a too strong ionization. As the *repeat* background model is computationally more demanding due to the spin polarization needed for several of the structures, we test if the “relaxed” background ionization effect can be approximated by simply scaling down the single particle PES computed with the *full* background model, with respective structure’s *q*_*Li*_ = *R* value. This *scaled* background model shows very similar results for all sets of *q*_*Li*_ as the *repeat* background model predicting all *D*_*s*_ values close to the target agreement for all charge sets and may be a good and computationally feasible alternative to the *repeat* background model.

Another observation that can be made based on the validation data set is that for “magnetic” systems, i.e., containing Fe, Ni, or Mn, Ionic TuTraSt does not perform worse although spin-polarization or charge-localization is not taken into account. This may, however, change in partially lithiated systems, where the locations and clustering of Li ions and polarons can affect the energy significantly.

#### 4.2.4 Computational Efficiency

The distribution of the number of required DFT calculations in the single-particle grid calculations and the number of steps in the AIMD simulations are shown for all structures in Figure 10.

**Figure 10:**
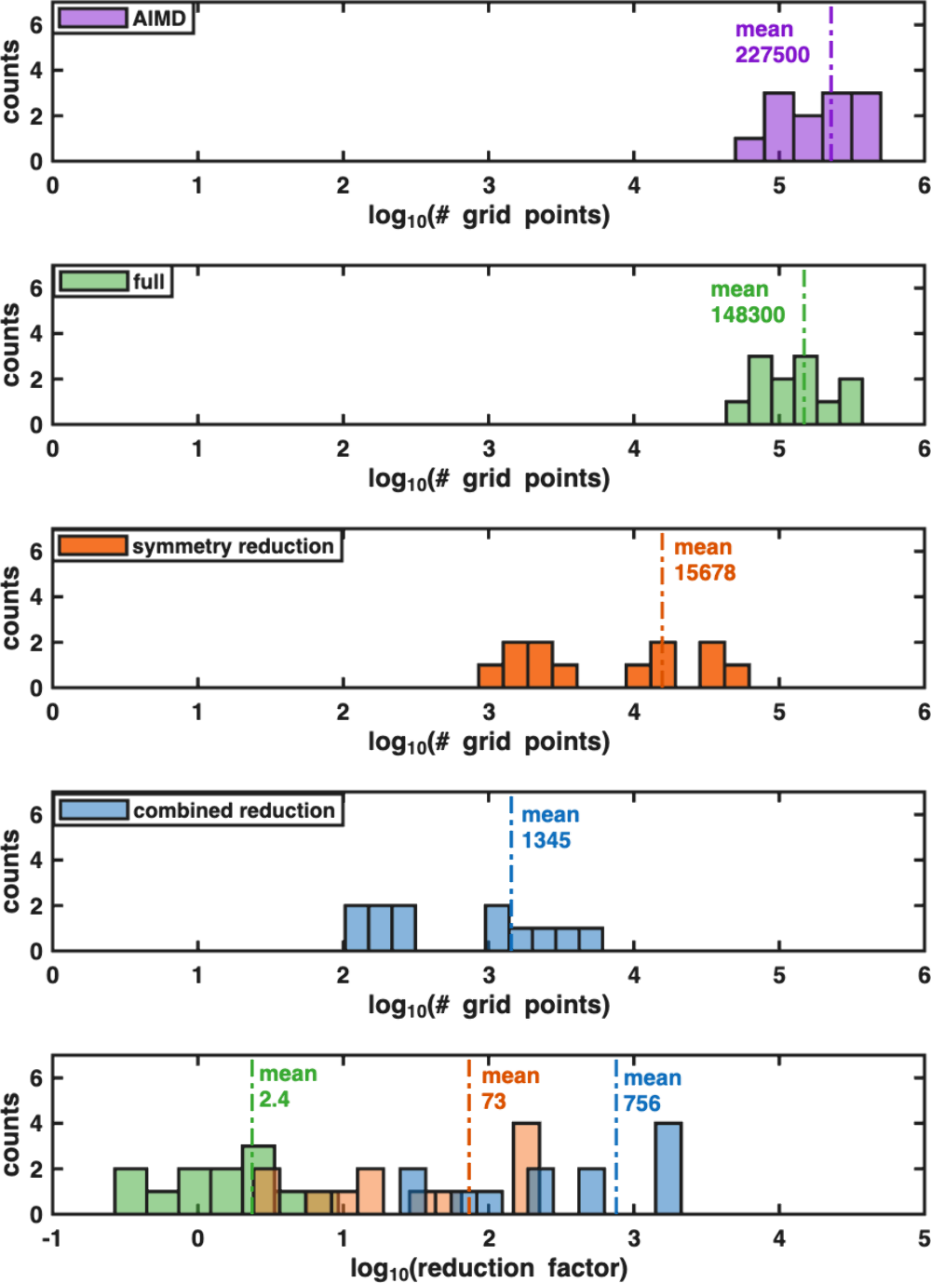
Distributions and means of the number of DFT single point energy calculations required for computing the self-diffusion coefficients for the data set of fast Li conductors using AIMD simulations (purple bars) vs the different sampling strategies for the Ionic TuTraSt approach. Full sampling (green bars) involves sampling of the single-particle potential energy surface without using any reduction strategies. Symmetry reduction (orange bars) excludes sampling of symmetry equivalent grid points. Combined reduction (blue bars) combines symmetry reduction with the optimized parameters for tricubic interpolation (grid resolution of 0.4 Å) and high energy exclusion (vdW scaling factor of 0.7).

This serves as an approximate comparison between the computational costs of the methods since the DFT calculations in the two methods are comparable and the MMC routine adds negligibly to the total computational cost in the DFT based Ionic TuTraSt routine. Comparing the number of DFT calculations rather than CPU hours focuses the discussion of computational efficiency on the method rather than software choices and parallelization or HPC hardware, implementation, and efficiency. A comparison of the CPU hours for calculations performed in this study is provided in Table S1 and Figure S21. The number of AIMD simulation steps of this set of fast ion conducting structures, range from 0.5–5 × 10^5^, averaging at 2.2 × 10^5^ as shown in Figure 10. A full grid sampling without applying the grid sampling reduction we present here would require an average of 1.4 × 10^5^ grid calculations resulting in only a small reduction of explicit DFT energy calculations with a structure average reduction factor of 2.4. Utilizing symmetry offers an already significant reduction factor of 73 relative AIMD and applying a vdW exclusion factor of 0.7 and sample at a 0.4 Å grid in combination with the tricubic interpolation scheme, the number of explicit grid calculations per structure ranges from 10^2^–10^4^ averaging at only 1345. This decreases the average number of calculations per structure by a factor of 756 compared to AIMD.

It should also be noted that these reductions are not representative of the practical reduction during, e.g., a screening procedure. These highly diffusive structures are chosen for validation purposes to be able to compare with AIMD. The fast diffusion allows the diffusive time scale to be reached in the AIMD simulations, in the given number of timesteps. However, for a general structure, the diffusion will be lower, and the diffusive time scale would be longer. An added benefit with the DFT-grid Ionic TuTraSt approach is that even if the diffusive time scale increases, the computational cost would remain the same, since the grid sampling is not dependent on the time evolution. For AIMD on the other hand, this would require longer simulations to obtain reliable predictions. In this view, the distribution of reduction factors for this set of high-diffusive structures in Figure 10 represents a lower bound to the general case. Overall, the grid sampling with the reduction strategies enables a reduction of 1.5–3.5 orders of magnitude compared to AIMD, enough to permit sampling the single-particle potential energy with DFT for diffusion prediction with Ionic TuTraSt. Since the accuracy compared to AIMD is maintained within 1 order of magnitude in the majority of cases, the reduction in computational cost can be argued to outweigh the loss in accuracy. This renders DFT-grid Ionic TuTraSt a good option for DFT-based diffusion prediction, for larger numbers of structures and at longer time scales, compared to AIMD.

Moreover, of the studied structures, the accurate predictions of the well-known superionic conductors Li_10_GeP_2_S_12_, Li_1.3_Al_0.3_Ti_1.7_(PO_4_)_3_, and Li_7_La_3_Zr_2_O_12_ are of particular interest. In 2017, Mo et al.^22^ were the first to attribute fast diffusion of these materials to the ions moving simultaneously in a liquid like and time dependent chain motion—a so-called concerted motion. As Ionic TuTraSt includes the interactions between ions through an effective field type model, time-correlations of such concerted motion processes will not be captured. However, the Ionic TuTraSt method, in fact, does reproduce accurate diffusion coefficients and energy landscapes for these studied structures compared with AIMD and also the observed decrease in activation energy with ion loading. This indicates that this is an effect explained by the contributions to the free energy from the configurational entropy of the interacting mobile Li-ions rather than that they necessarily need to diffuse concertedly in a time-dependent manner. This is discussed further in our parallel paper.^47^

## 5 Conclusion

By leveraging space-group symmetries, a tricubic interpolation scheme, and excluding high-energy atomic overlap regions, we demonstrate that the number of grid point calculations required to construct a potential energy grid for a Li-ion in a ceramic Li-ion conductor can typically be reduced by 2–3 orders of magnitude without significant loss of accuracy. This extensive reduction of explicit energy calculations required to construct such a grid makes feasible the use of higher level theory calculations, i.e., DFT.

With such a DFT-based single-particle grid as input for our previously developed Ionic TuTraSt method, the accuracy of prediction relative to using classical force fields and transferability for computing Li-ion diffusion coefficients within the large chemical space of ceramic electrolytes are significantly improved.

We further show that self-diffusion coefficients computed with DFT-grid Ionic TuTraSt for 14 known superionic conductors have an excellent correlation with those computed by ab initio molecular dynamics. This provides proof that the low energy barriers seen in ceramic superionic conductors can be described by an effective field model to include ion–ion configurational entropy without the need for time-dependent correlation.

The reduced computational cost that the DFT-grid Ionic TuTraSt method entails to only hundreds to thousands of DFT calculations per structure rather than hundreds-of-thousands, such as typically needed at the very least in AIMD, realizes the possibility of carrying out large-scale screening studies of extended sets of structures. Our method also expands the lower range of diffusion coefficient that can be computed without increasing the computational cost or suffering from convergence issues such as in AIMD. Considering that only those structures among the fastest known ion conductors were tested, this sets a lower limit to potential reduction computational cost relative to studying a set of materials with a broader range of ion diffusivity where the expected cost reduction will scale with the same order as the decrease of diffusion rates.

The validation toward AIMD data also showed that constructing the single-particle DFT grid with a background charge adapted to the structure-specific Li-ionization values predicted by the repeat model improved the stability of prediction by DFT-grid Ionic TuTraSt relative to AIMD. The most stable results were achieved together with structure specific REPEAT point charges used to model Li–Li Coulomb interactions during the Metropolis Monte Carlo step of the Ionic TuTraSt workflow as did a constant point charge of *q*_*Li*_ = 0.5*e* such as shown in our previous work.^11^ An interesting and somewhat counterintuitive observation is that even the complete exclusion of Coulomb interactions (*q*_*Li*_ = 0*e*) still gave stable results for all but one false positive structure. This indicates that including only a vdW potential is sufficient to effectively capture the short-range ion repulsions in most structures.

The presented strategies that comprise the DFT-grid Ionic TuTraST approach make possible predictions of diffusion coefficients at a DFT-level accuracy in close correspondence to the performance of AIMD, but at a computational cost that can allow the study of a large number of different structures extending through a much larger volume of the vast computational space of ceramic ion conductors than what is possible today. Furthermore, it is conceivable that the reduction strategies presented here can be further developed to reduce the number of explicit energy calculations needed, such as more advanced interpolation schemes and strategies to identify and exclude improbable high-energy regions of the PES. We anticipate that machine learning algorithms will play an important role here. For example, in a parallel study, we are investigating the use of foundational models^48,49^ showing promise of accurately reproducing of DFT-computed PES grids trained on only a small portion of the grid data.

## Supplementary Material



## Notes

Additional computational details and analysis results, including: symmetry and space group distributions; AIMD mean square displacement plots; results from convergence tests; CPU hour comparison between methods; and Li_6_PS_5_Cl potential energy surfaces (PDF)

## References

[1] Schwarz F., Barthel S., Mace A. (2024). Understanding Mobile Particles in
Solid-State Materials: From the Perspective of Potential Energy Surfaces. Chem. Mater..

[2] Jónsson, H. , Mills, G. , Jacobsen, K. W. Nudged elastic band method for finding minimum energy paths of transitions In Classical and Quantum Dynamics in Condensed Phase Simulations; World Scientific: 1998; pp 385–404, 10.1142/9789812839664_0016.

[3] Altundal O. F., Altintas C., Keskin S. (2020). Can COFs replace
MOFs in flue gas
separation? high-throughput computational screening of COFs for CO2/N2
separation. J. Mater. Chem. A.

[4] Mace A., Barthel S., Smit B. (2019). Automated
Multiscale Approach To
Predict Self-Diffusion from a Potential Energy Field. J. Chem. Theory Comput..

[5] Kim J., Abouelnasr M., Lin L.-C., Smit B. (2013). Large-Scale Screening
of Zeolite Structures for CO2Membrane Separations. J. Am. Chem. Soc..

[6] Ren E., Coudert F.-X. (2023). Enhancing Gas Separation Selectivity Prediction through
Geometrical and Chemical Descriptors. Chem.
Mater..

[7] Adams S. (2024). Origin of
Fast Li+-Ion Conductivity in the Compressible Oxyhalide LiNbOCl4. Energy Storage Mater..

[8] Wong L. L., Phuah K. C., Dai R., Chen H., Chew W. S., Adams S. (2021). Bond Valence Pathway
Analyzer–An Automatic Rapid Screening
Tool for Fast Ion Conductors within softBV. Chem. Mater..

[9] Chen H., Wong L. L., Adams S. (2019). *SoftBV* –
a software tool for screening the materials genome of inorganic fast
ion conductors. Acta Crystallogr. B.

[10] Nakayama M., Kimura M., Jalem R., Kasuga T. (2016). Efficient automatic
screening for Li ion conductive inorganic oxides with bond valence
pathway models and percolation algorithm. Jpn.
J. Appl. Phys..

[11] Gustafsson H., Kozdra M., Smit B., Barthel S., Mace A. (2024). Predicting
Ion Diffusion from the Shape of Potential Energy Landscapes. J. Chem. Theory Comput..

[12] Voter, A. F. Introduction to the kinetic Monte Marlo method. In Radiation Effects in Solids; Sickafus, K. E. , Kotomin, E. A. , Uberuaga, B. P. , Eds.; Springer: Dordrecht, 2007; Vol. 235; pp 1–23.

[13] Deng Z., Mishra T. P., Mahayoni E., Ma Q., Tieu A. J. K., Guillon O., Chotard J.-N., Seznec V., Cheetham A. K., Masquelier C., Gautam G. S., Canepa P. (2022). Fundamental
investigations
on the sodium-ion transport properties of mixed polyanion solid-state
battery electrolytes. Nat. Commun..

[14] Zhu L., Wang Y., Chen J., Li W., Wang T., Wu J., Han S., Xia Y., Wu Y., Wu M., Wang F., Zheng Y., Peng L., Liu J., Chen L., Tang W. (2022). Enhancing ionic conductivity in solid
electrolyte by relocating diffusion ions to under-coordination sites. Sci. Adv..

[15] Sjølin B. H., Jørgensen P. B., Fedrigucci A., Vegge T., Bhowmik A., Castelli I. E. (2023). Accelerated Workflow for Antiperovskite-based Solid
State Electrolytes. Batter Supercaps.

[16] Mace A., Leetmaa M., Laaksonen A. (2015). Temporal Coarse
Graining of CO2 and
N2 Diffusion in Zeolite NaKA: From the Quantum Scale to the Macroscopic. J. Chem. Theory Comput..

[17] Bachman J. C., Muy S., Grimaud A., Chang H.-H., Pour N., Lux S. F., Paschos O., Maglia F., Lupart S., Lamp P., Giordano L., Shao-Horn Y. (2016). Inorganic
Solid-State Electrolytes
for Lithium Batteries: Mechanisms and Properties Governing Ion Conduction. Chem. Rev..

[18] Rappe A. K., Casewit C. J., Colwell K. S., Goddard W. A. I., Skiff W. M. (1992). UFF, a
full periodic table force field for molecular mechanics and molecular
dynamics simulations. J. Am. Chem. Soc..

[19] Dubbeldam D., Calero S., Ellis D. E., Snurr R. Q. (2016). RASPA: molecular
simulation software for adsorption and diffusion in flexible nanoporous
materials. Mol. Simul..

[20] Lekien F., Marsden J. (2005). Tricubic interpolation
in three dimensions. Int. J. Numer. Meth. Engng.

[21] Talirz L., Kumbhar S., Passaro E., Yakutovich A. V., Granata V., Gargiulo F., Borelli M., Uhrin M., Huber S. P., Zoupanos S., Adorf C. S., Andersen C. W., Schütt O., Pignedoli C. A., Passerone D., VandeVondele J., Schulthess T. C., Smit B., Pizzi G., Marzari N. (2020). Materials Cloud, a
platform for open computational
science. Sci. Data.

[22] He X., Zhu Y., Mo Y. (2017). Origin of fast ion diffusion in super-ionic
conductors. Nat. Commun..

[23] Weber D. A., Senyshyn A., Weldert K. S., Wenzel S., Zhang W., Kaiser R., Berendts S., Janek J., Zeier W. G. (2016). Structural
Insights and 3D Diffusion Pathways within the Lithium Superionic Conductor
Li10GeP2S12. Chem. Mater..

[24] Burbano M., Carlier D., Boucher F., Morgan B. J., Salanne M. (2016). Sparse Cyclic
Excitations Explain the Low Ionic Conductivity of Stoichiometric Li_7_La_3_Zr_2_O_12_. Phys. Rev. Lett..

[25] Kirsch C., Dreßler C., Sebastiani D. (2022). Atomistic Diffusion Pathways of Lithium
Ions in Crystalline Lithium Silicides from ab Initio Molecular Dynamics
Simulations. J. Phys. Chem. C.

[26] Chen D., Jie J., Weng M., Li S., Chen D., Pan F., Wang L.-W. (2019). High throughput
identification of Li ion diffusion
pathways in typical solid state electrolytes and electrode materials
by BV-Ewald method. J. Mater. Chem. A.

[27] Yin Y.-C., Yang J.-T., Luo J.-D., Lu G.-X., Huang Z., Wang J.-P., Li P., Li F., Wu Y.-C., Tian T., Meng Y.-F., Mo H.-S., Song Y.-H., Yang J.-N., Feng L.-Z., Ma T., Wen W., Gong K., Wang L.-J., Ju H.-X., Xiao Y., Li Z., Tao X., Yao H.-B. (2023). A LaCl3-based lithium superionic
conductor compatible with lithium metal. Nature.

[28] Baktash A., Reid J. C., Roman T., Searles D. J. (2020). Diffusion of lithium
ions in Lithium-argyrodite solid-state electrolytes. npj Comput. Mater..

[29] Zhou J., Chen P., Wang W., Zhang X. (2022). Li7P3S11 electrolyte
for all-solid-state lithium-ion batteries: structure, synthesis, and
applications. Chem. Eng. J..

[30] Zhang J., Dong Y., Wang C.-A. (2024). Surface-Like Diffusion
of Fast Ions
in Framework Energy Materials for Li- and Na-Ion Batteries. Angew. Chem., Int. Ed.

[31] Setyawan W., Curtarolo S. (2010). High-throughput electronic band structure calculations:
Challenges and tools. Comput. Mater. Sci..

[32] Ong S. P., Richards W. D., Jain A., Hautier G., Kocher M., Cholia S., Gunter D., Chevrier V. L., Persson K. A., Ceder G. (2013). Python Materials Genomics
(pymatgen): A robust, open-source python
library for materials analysis. Comput. Mater.
Sci..

[33] Togo A., Shinohara K., Tanaka I. (2024). Spglib: a software library for crystal
symmetry search. Sci. Technol. Adv. Mater.,
Meth..

[34] Wojdyr M. (2022). GEMMI: A library
for structural biology. J. Open Source Softw..

[35] Mentel, Ł. mendeleev – A Python package with properties of chemical elements, ions, isotopes and methods to manipulate and visualize periodic table. 2021; 10.5281/zenodo.7373791.

[36] Kühne T. D., Iannuzzi M., Del Ben M., Rybkin V. V., Seewald P., Stein F., Laino T., Khaliullin R. Z., Schütt O., Schiffmann F., Golze D., Wilhelm J., Chulkov S., Bani-Hashemian M. H., Weber V., Borštnik U., Taillefumier M., Jakobovits A. S., Lazzaro A., Pabst H., Müller T., Schade R., Guidon M., Andermatt S., Holmberg N., Schenter G. K., Hehn A., Bussy A., Belleflamme F., Tabacchi G., Glöß A., Lass M., Bethune I., Mundy C. J., Plessl C., Watkins M., VandeVondele J., Krack M., Hutter J. (2020). CP2K: An electronic
structure and molecular dynamics software package - Quickstep: Efficient
and accurate electronic structure calculations. J. Chem. Phys..

[37] Perdew J. P., Burke K., Ernzerhof M. (1996). Generalized
Gradient Approximation
Made Simple. Phys. Rev. Lett..

[38] Grimme S., Antony J., Ehrlich S., Krieg H. (2010). A consistent and accurate
ab initio parametrization of density functional dispersion correction
(DFT-D) for the 94 elements H-Pu. J. Chem. Phys..

[39] Grimme S., Ehrlich S., Goerigk L. (2011). Effect of
the damping function in
dispersion corrected density functional theory. J. Comput. Chem..

[40] Lu J.-B., Cantu D. C., Nguyen M.-T., Li J., Glezakou V.-A., Rousseau R. (2019). Norm-Conserving Pseudopotentials and Basis Sets To
Explore Lanthanide Chemistry in Complex Environments. J. Chem. Theory Comput..

[41] VandeVondele J., Hutter J. (2007). Gaussian basis sets for accurate
calculations on molecular
systems in gas and condensed phases. J. Chem.
Phys..

[42] Goedecker S., Teter M., Hutter J. (1996). Separable
dual-space Gaussian pseudopotentials. Phys.
Rev. B.

[43] Hartwigsen C., Goedecker S., Hutter J. (1998). Relativistic separable dual-space
Gaussian pseudopotentials from H to Rn. Phys.
Rev. B.

[44] Krack M. (2005). Pseudopotentials
for H to Kr optimized for gradient-corrected exchange-correlation
functionals. Theor. Chem. Acc..

[45] Campañá C., Mussard B., Woo T. K. (2009). Electrostatic Potential Derived Atomic
Charges for Periodic Systems Using a Modified Error Functional. J. Chem. Theory Comput..

[46] Kahle L., Marcolongo A., Marzari N. (2018). Modeling lithium-ion solid-state
electrolytes with a pinball model. Phys. Rev.
Mater..

[47] Schwarz, F. ; Gustafsson, H. ; Mace, A. Investigating configurational entropy in ceramic super-ionic conductors. 2025, Manuscript in preparation.

[48] Batatia, I. ; Kovacs, D. P. ; Simm, G. ; Ortner, C. ; Csanyi, G. MACE: Higher Order Equivariant Message Passing Neural Networks for Fast and Accurate Force Fields. Advances in Neural Information Processing Systems; 2022; pp 11423–11436.

[49] Deng B., Zhong P., Jun K., Riebesell J., Han K., Bartel C. J., Ceder G. (2023). CHGNet as a pretrained universal
neural network potential for charge-informed atomistic modelling. Nat. Mach. Intell..

