# Supplementary material for: Computationally Efficient DFT-Based Sampling of Ion Diffusion in Crystalline Solids
Source: J Chem Theory Comput. 2025 Sep 3;21(18):8669–82. doi: 10.1021/acs.jctc.5c00891 (PMC12461913; doi:10.1021/acs.jctc.5c00891)
Supplement: Supplementary file 1 [file ct5c00891_si_001.pdf]

Supporting Information: Computationally Efficient DFT-based Sampling of Ion Diffusion in Crystalline Solids

Hannes Gustafsson,† Fabian Schwarz,† Thijs Smolders,† Senja Barthel,‡ and Amber Mace*,†

†*Department of Chemistry – Ångström, Uppsala University, SE-751 21 Uppsala, Sweden*

‡*Department of Mathematics, Vrije Universiteit, 1081 HV Amsterdam, Netherlands*

E-mail: amber.mace@kemi.uu.se

Symmetry and Space Group Distributions

The space group distribution of the dataset is given in Figure S1, and the distribution of the inverse reduction factor, i.e. the fraction of the number of grid points after symmetry reduction over the total number of grid points, is plotted for all space groups for different mesh sizes, assuming equally many points in each direction.

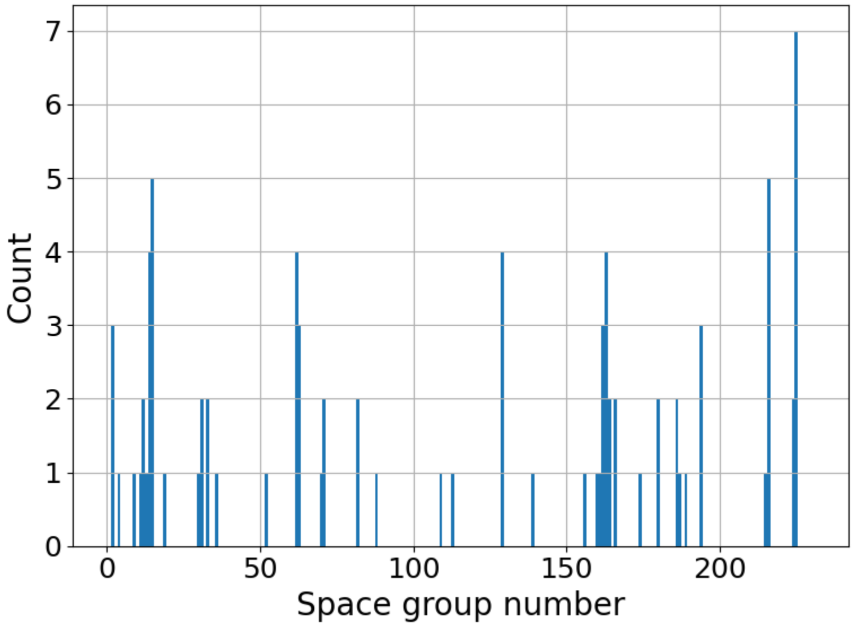

Figure S1: The distribution of space groups among the test set of 84 structures. The international space group numbers correspond to the International Tables.

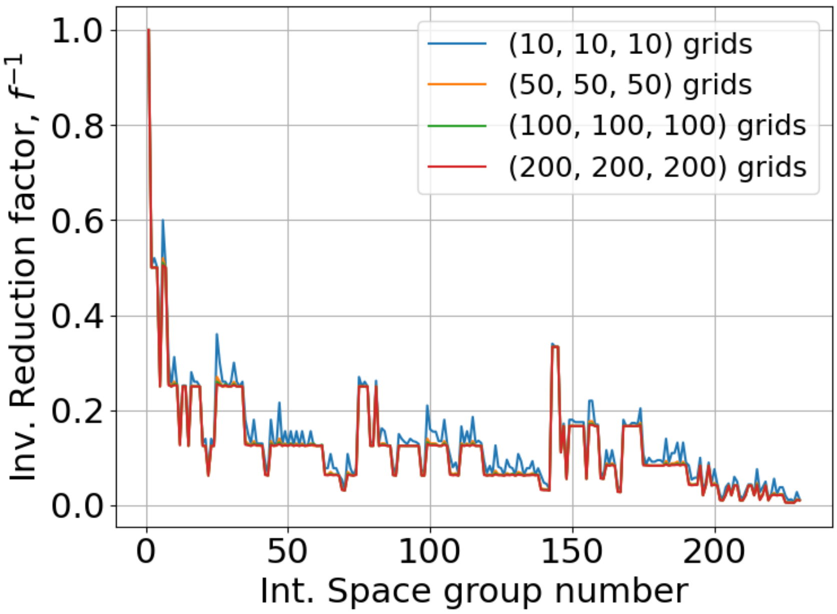

Figure S2: Distribution of the inverse reduction factor due to symmetry, defined as the number of symmetry-unique points divided by the total number of grid points, for isotropic grids of varying density. Results are plotted for all space groups in their reference setting in the space group table in gemmi.

Non-orthogonal Grids

In RASPA,[S1] energy grids, including their derivatives, are internally saved in orthogonal grids. Additionally, all grid points on the border of the unit cell are explicitly saved in all equivalent points on the border(in 3 dimensions, the origin is saved 8 times, all other points lying on an edge are saved 4 times, and all remaining points lying on a face are saved twice). When evaluating the energy at a specific location, the point is first mapped into the original unit cell. The energy is then calculated by interpolating over the 8 corners of the respective cube in the internal RASPA grid. Figure S3 illustrates in 2 dimensions how a non-orthogonal grid is mapped onto the internal RASPA grid.

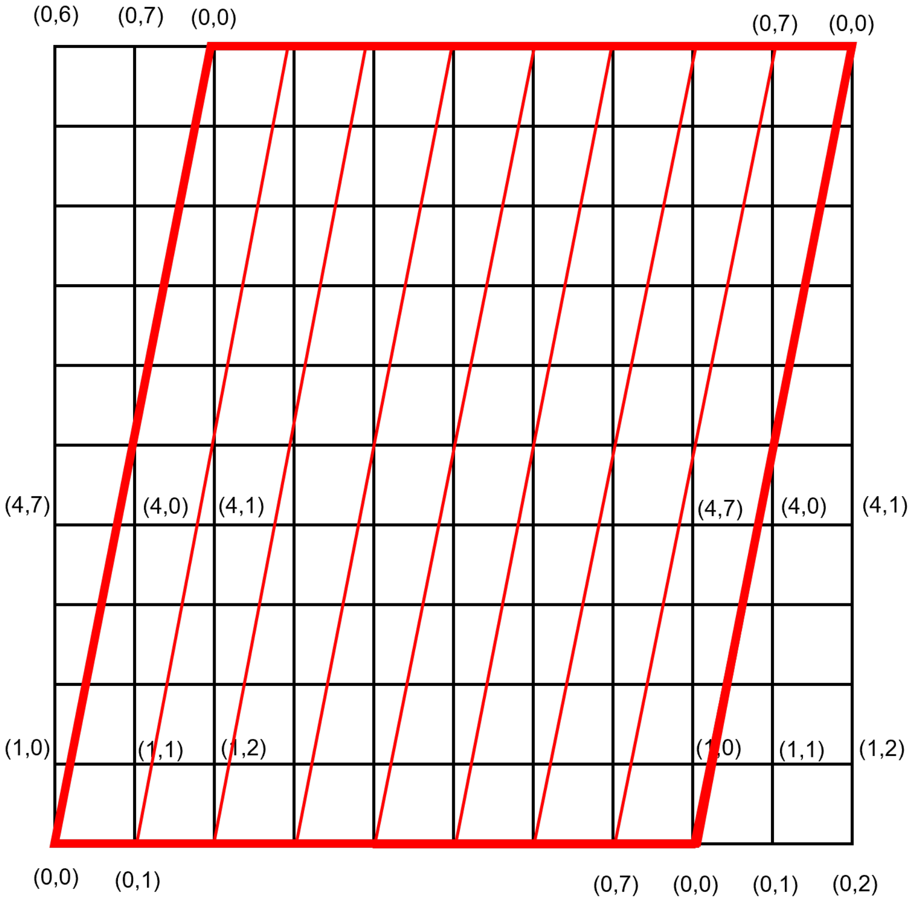

Figure S3: Sketch illustrating how non-orthogonal single-particle grids are mapped to an orthogonal RASPA grid. Here (i, j) refers to the indices of the original, non-orthogonal grid. Each original grid point is mapped to the closest grid point on the orthogonal RASPA grid. Grid points in the RASPA grid, which lie outside of the unit cell, are padded accordingly.

AIMD Mean Square Displacement Analysis and Computed Self Diffusion Coefficients

Mean square displacements (MSD) from the ab initio molecular dynamics simulations carried out are presented for each structure on both the linear scale (right panels) and the log-log scale (left panels) in figures S4−S11. Blue lines show the overall three dimensional MSD while the orange, yellow and purple lines show the one dimensional MSD along the respective cell basis vectors a, b and c. The diffusion coefficient is computed from the slope of 100 points of the three dimensional MSD data defined as the fitting window and marked in green.

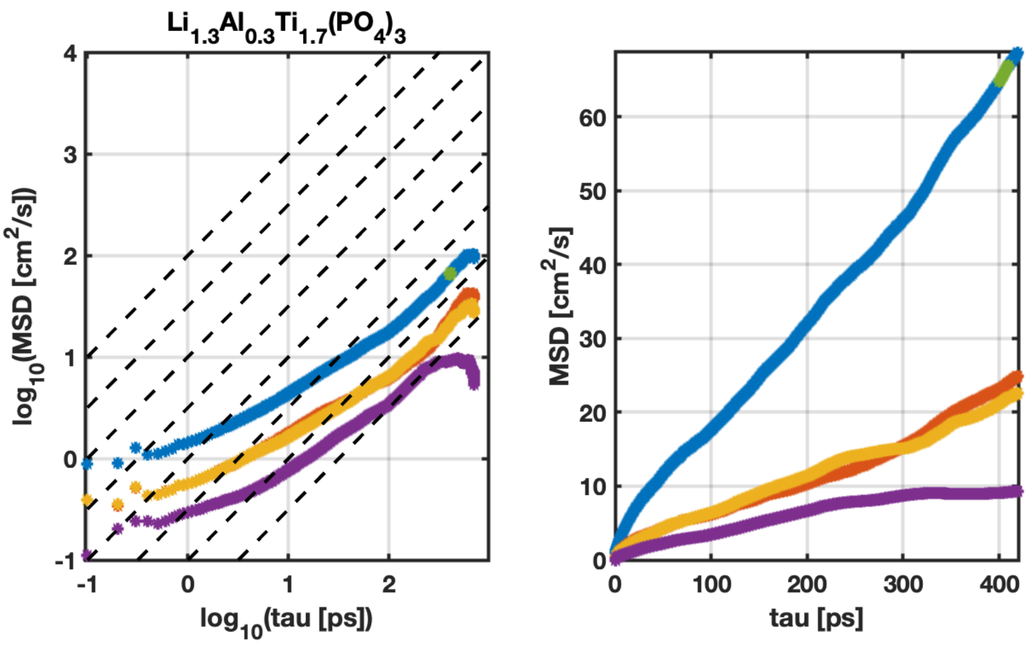

Figure S4: Ab initio molecular dynamics mean square displacements of Li in $Li_{1.3}Al_{0.3}Ti_{1.7}(PO_4)_3$. Computed diffusion coefficient: $D_s = 4.1 \times 10^{-6} cm^2/s$.

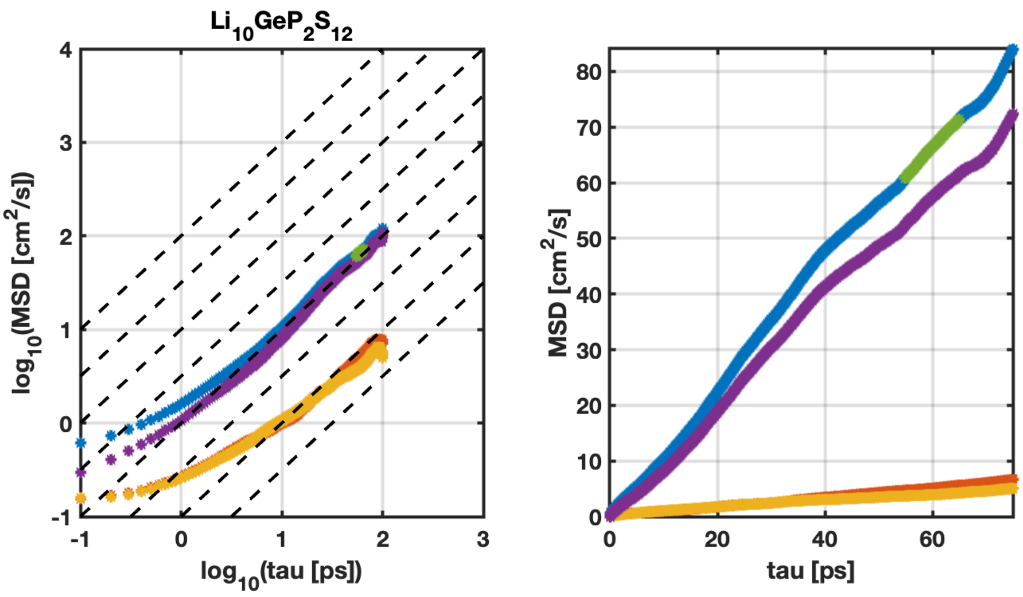

Figure S5: Ab initio molecular dynamics mean square displacements of Li in $Li_{10}GeP_2S_{12}$. Computed diffusion coefficient: $D_s = 1.7 \times 10^{-5} cm^2/s$.

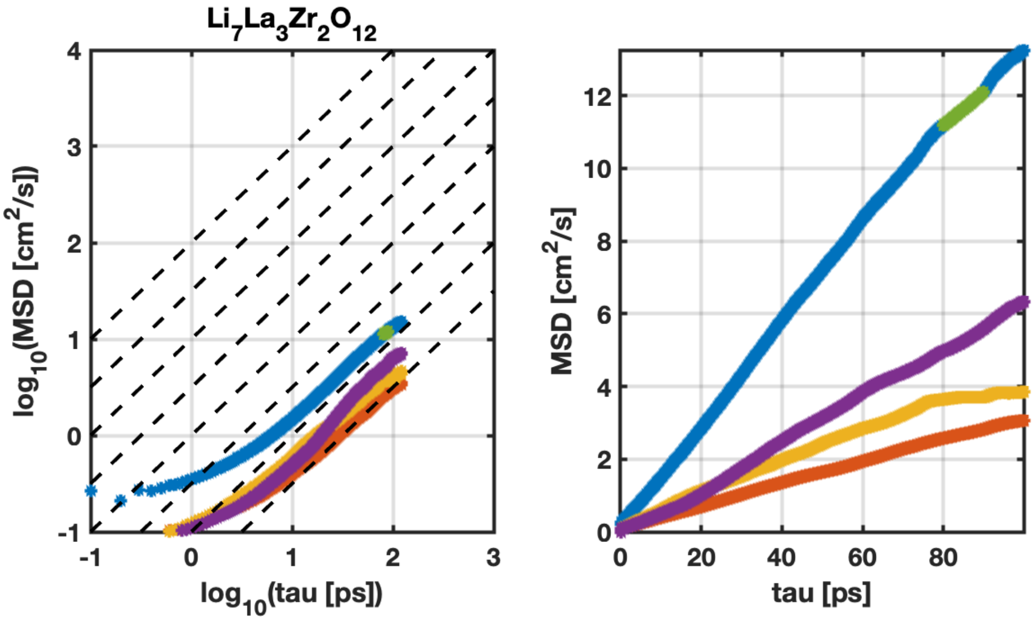

Figure S6: Ab initio molecular dynamics mean square displacements of Li in $Li_7La_3Zr_2O_{12}$. Computed diffusion coefficient: $D_s = 1.5 \times 10^{-6} cm^2/s$.

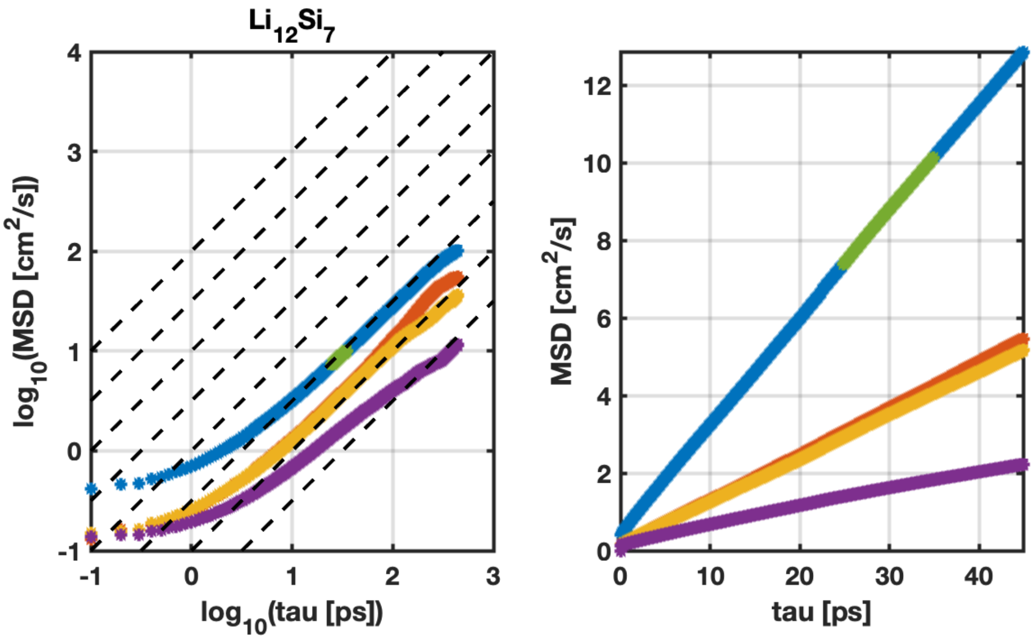

Figure S7: Ab initio molecular dynamics mean square displacements of Li in $Li_{12}Si_7$. Computed diffusion coefficient: $D_s = 4.6 \times 10^{-6} cm^2/s$.

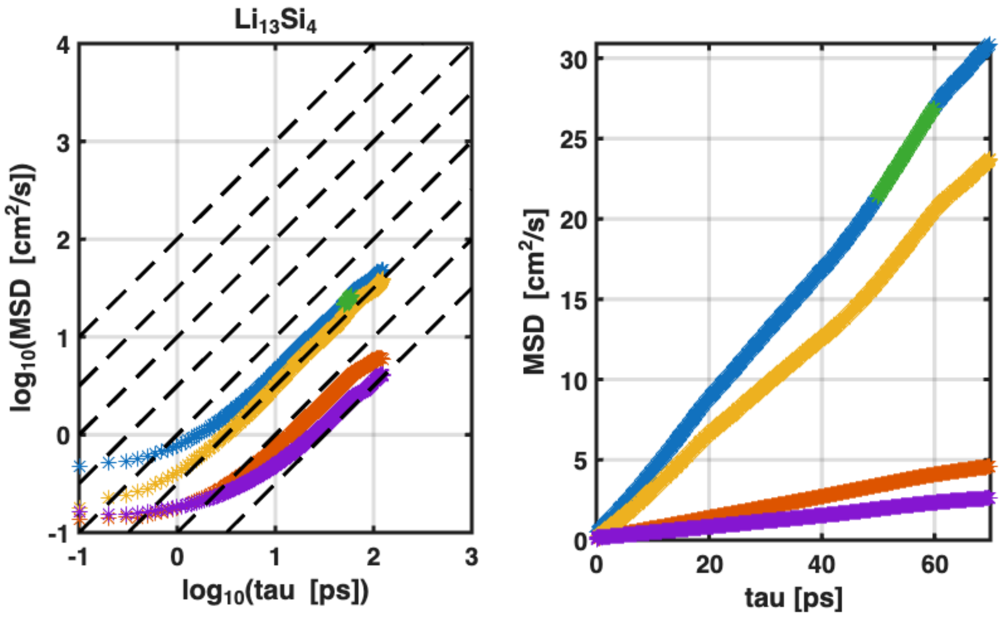

Figure S8: Ab initio molecular dynamics mean square displacements of Li in $Li_{13}Si_4$. Computed diffusion coefficient: $D_s = 9.2 \times 10^{-6} cm^2/s$.

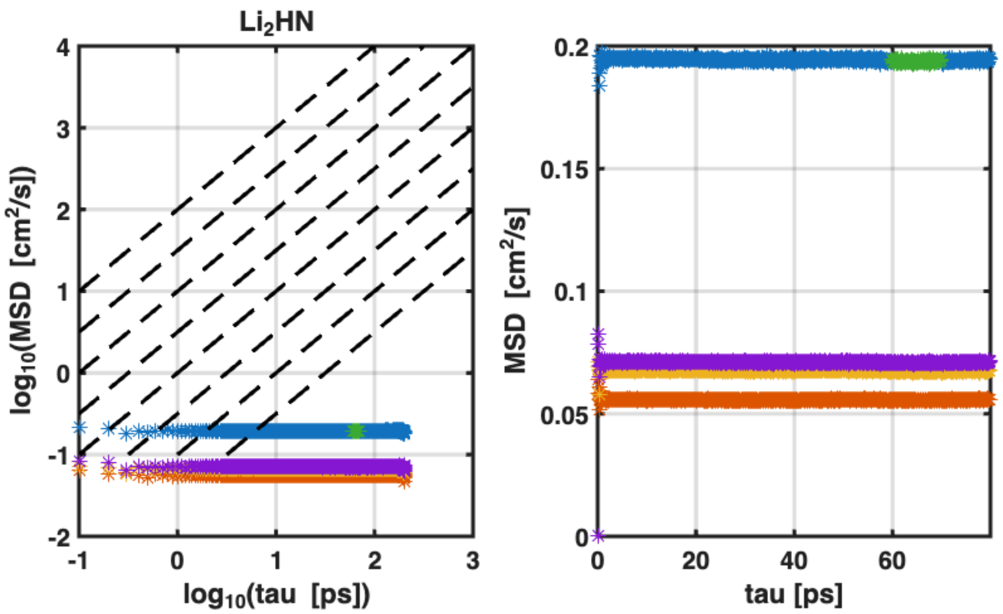

Figure S9: Ab initio molecular dynamics mean square displacements of Li in Li_2HN. No diffusion observed.

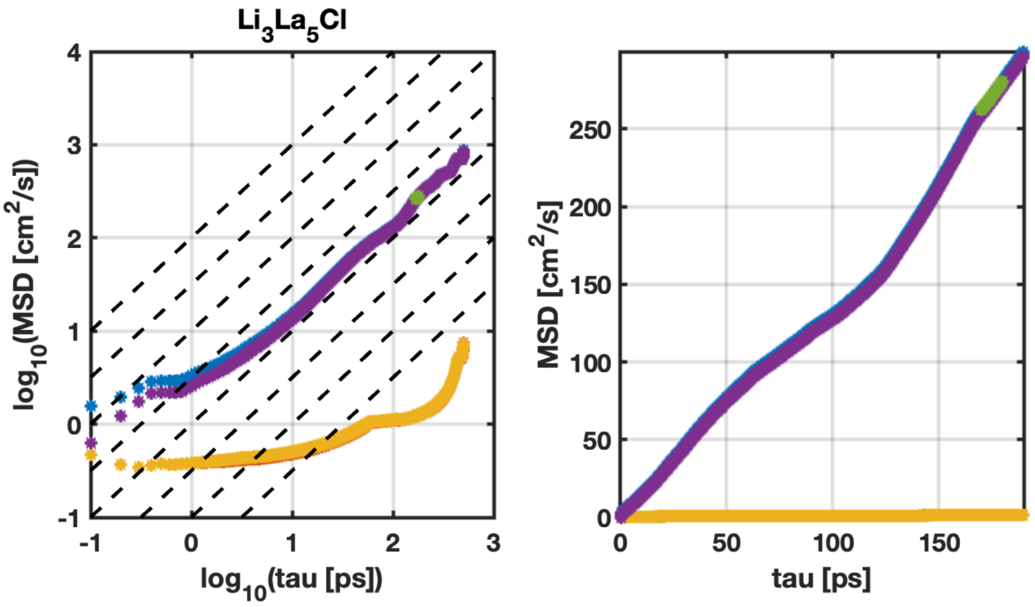

Figure S10: Ab initio molecular dynamics mean square displacements of Li in $Li_3La_5Cl_{18}$. Computed diffusion coefficient: $D_s = 3.1 \times 10^{-5} cm^2/s$.

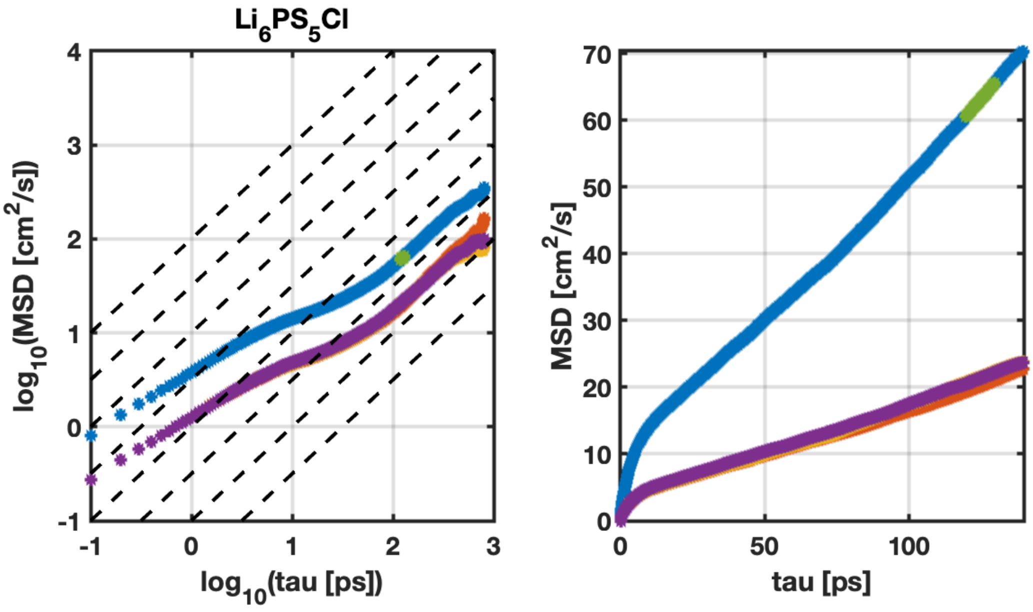

Figure S11: Ab initio molecular dynamics mean square displacements of Li in Li_6PS_5Cl. Computed diffusion coefficient: $D_s = 8.3 \pm 1.2 \times 10^{-6} cm^2/s$.

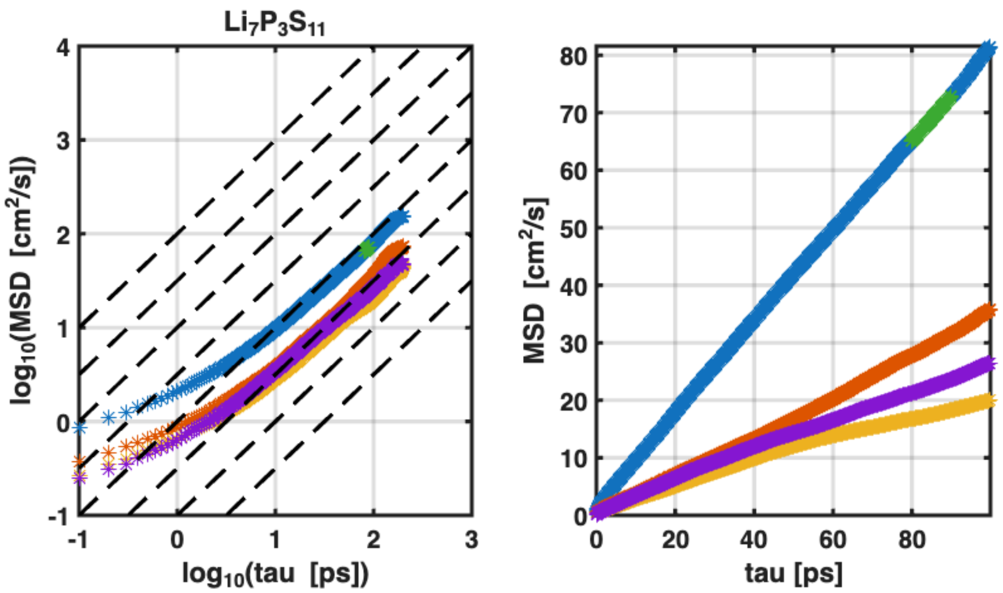

Figure S12: Ab initio molecular dynamics mean square displacements of Li in $Li_7P_3S_{11}$. Computed diffusion coefficient: $D_s = 1.3 \times 10^{-5} cm^2/s$.

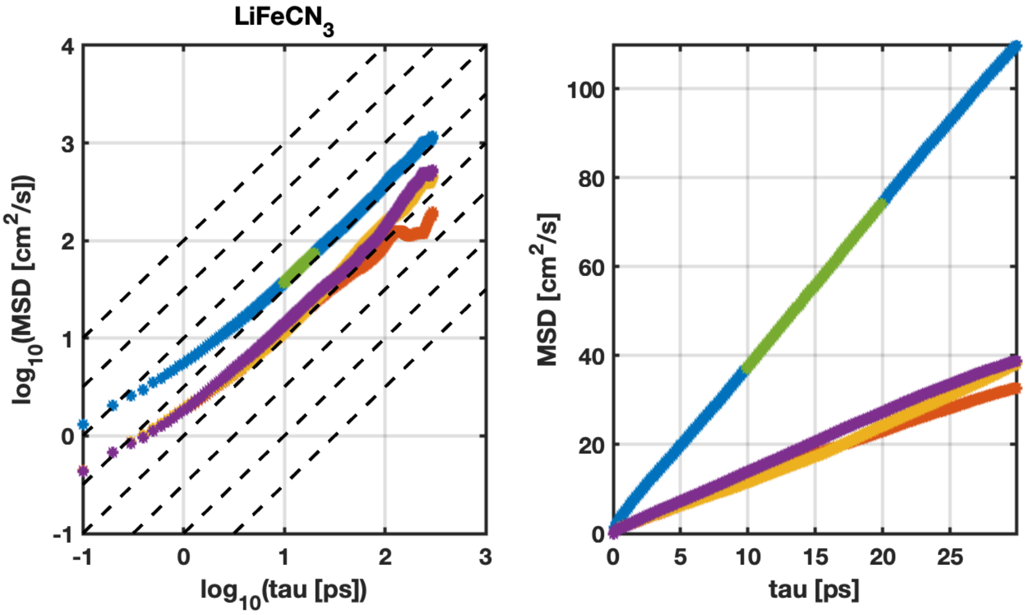

Figure S13: Ab initio molecular dynamics mean square displacements of Li in $LiFe(CN)_3$. Computed diffusion coefficient: $D_s = 6.2 \pm 3.2 \times 10^{-5} cm^2/s$.

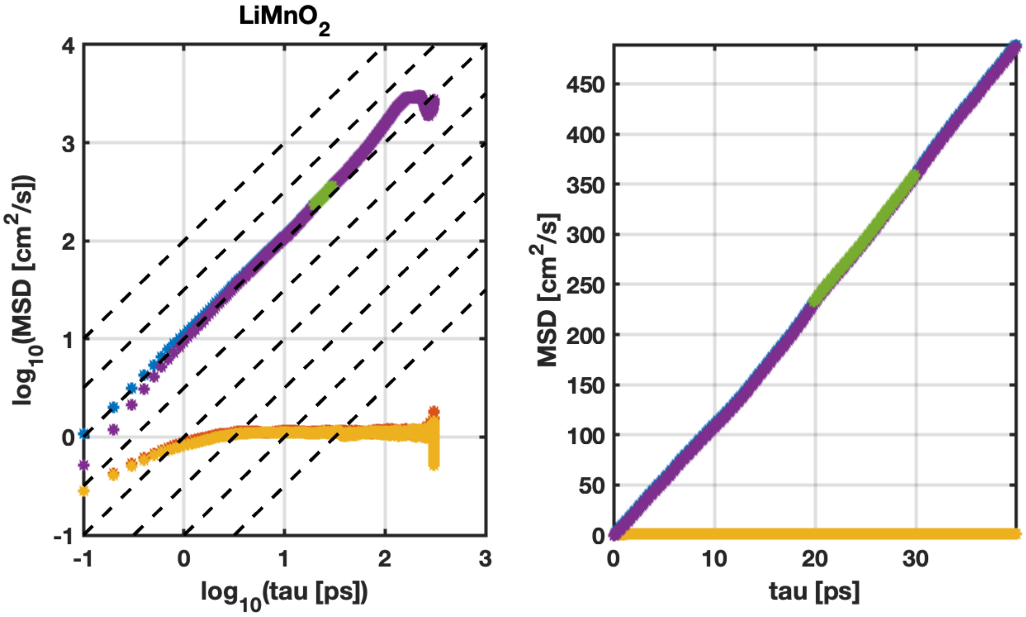

Figure S14: Ab initio molecular dynamics mean square displacements of Li in $LiMnO_2$. Computed diffusion coefficient: $D_s = 2.1e \times 10{-}4cm^2/s$.

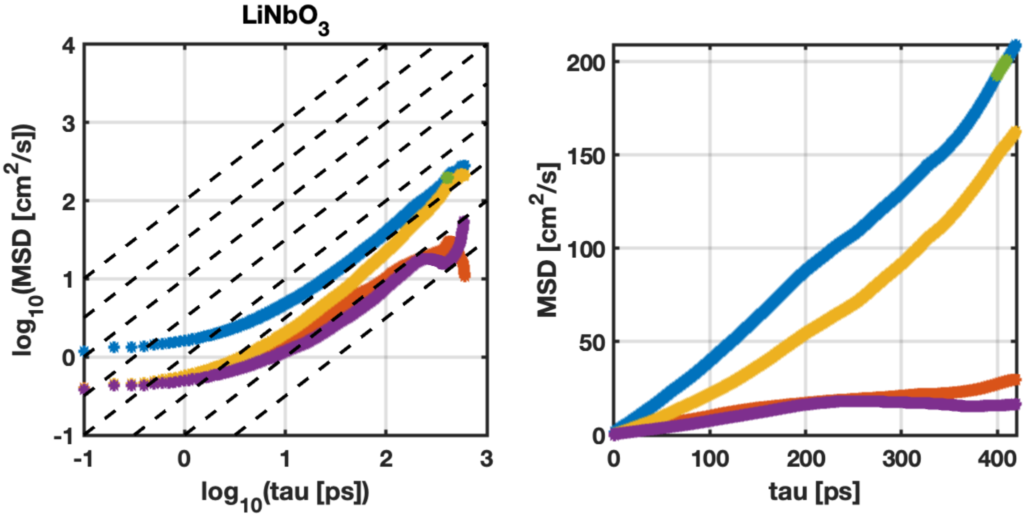

Figure S15: Ab initio molecular dynamics mean square displacements of Li in $LiNbO_3$. Computed diffusion coefficient: $D_s = 1.4 \pm 1.7e \times 10{-}5cm^2/s$.

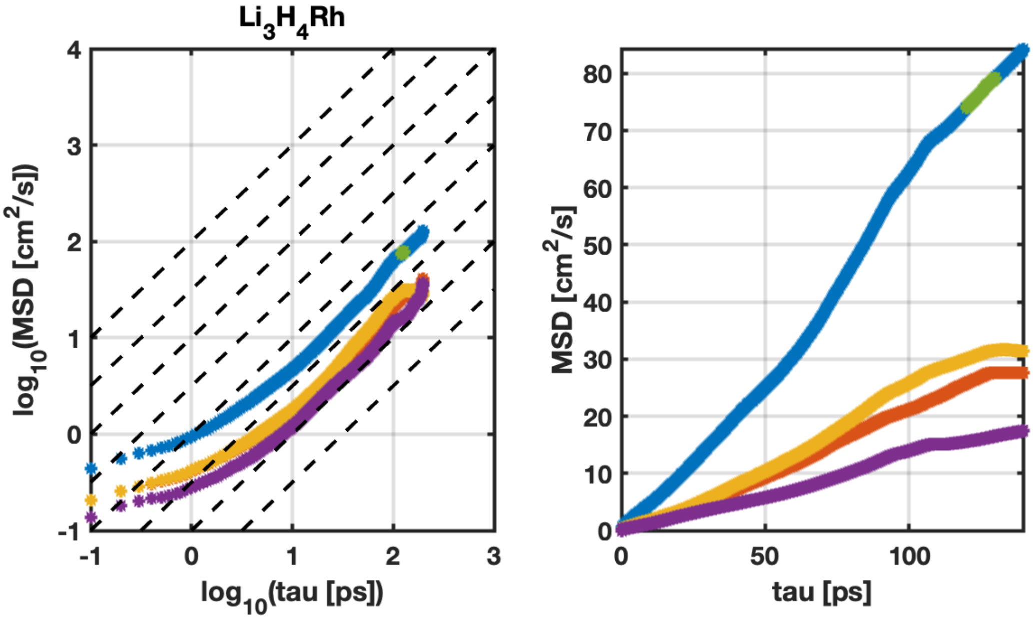

Figure S16: Ab initio molecular dynamics mean square displacements of Li in Li_3H_4Rh. Computed diffusion coefficient: $D_s = 8.9e \times 10{-}6cm^2/s$.

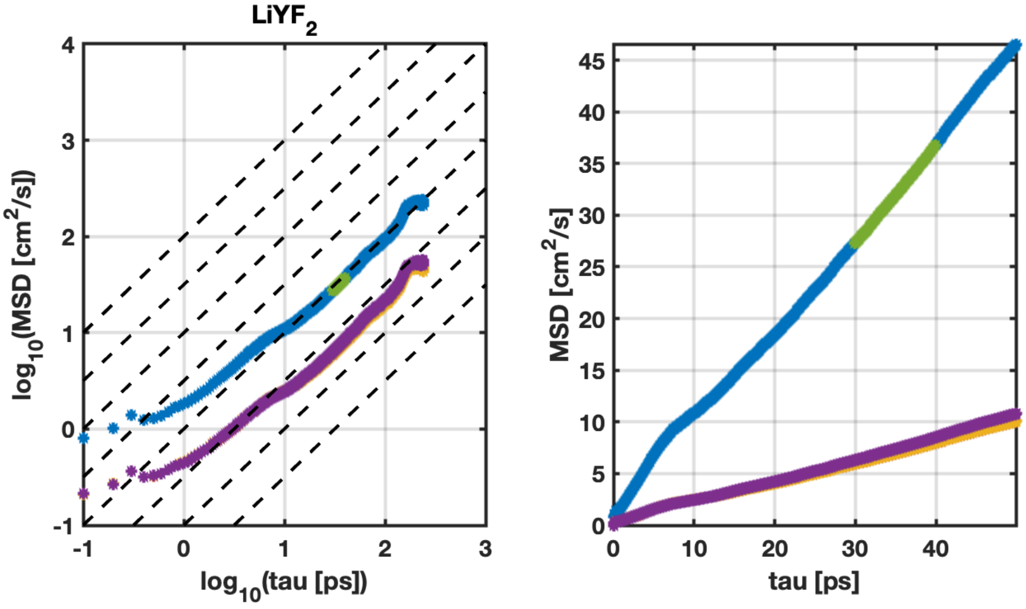

Figure S17: Ab initio molecular dynamics mean square displacements of Li in $LiYF_2$. Computed diffusion coefficient: $D_s = 1.6e \times 10{-}5cm^2/s$.

Convergence Tests

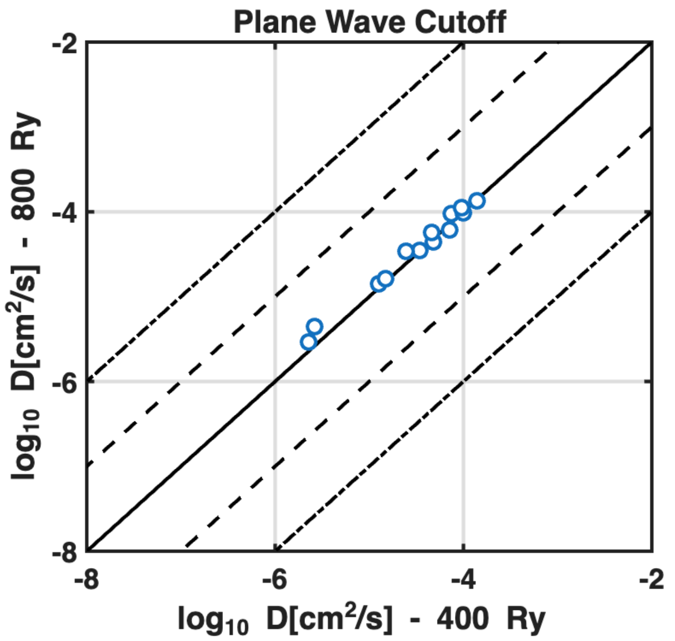

Figure S18: Comparison of diffusion coefficients predicted by Ionic TuTraST based on single-particle potential energy grid inputs computed with DFT using 400 Ry and 800 Ry plane wave cutoffs respectively for all structures in AIMD validations set.

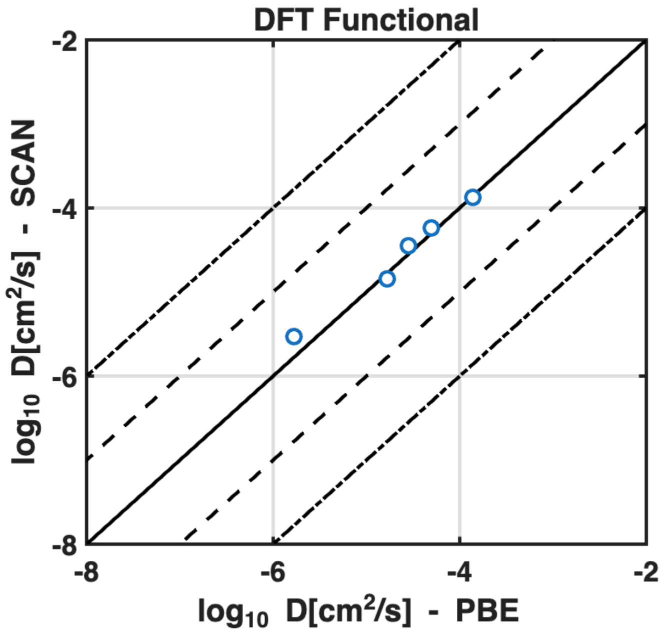

Figure S19: Comparison of diffusion coefficients predicted by Ionic TuTraST based on single-particle potential energy grid inputs computed with PBE+D3 and SCAN DFT functionals, respectively for structures Li_6PS_5Cl, $Li_7La_3Zr_2O_{12}$, $Li_{10}Ge(PS_6)$, $LiFe(CN)_3$ and $LiNbO_3$ selected This selection of structures was based on their low grid counts in order to mitigate the significantly higher computational costs involved with r2SCAN compared to PBE+D3.

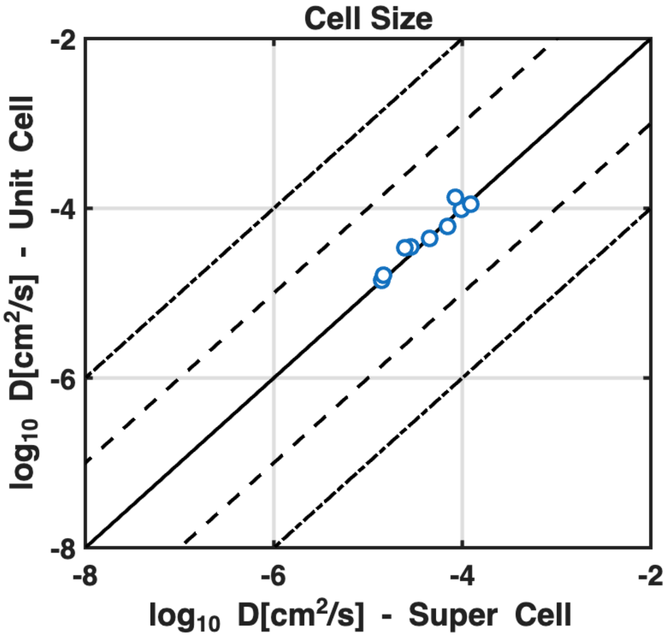

Figure S20: Comparison of diffusion coefficients predicted by Ionic TuTraST based on single-particle potential energy grid inputs computed on unit cells relative supercells fulfilling self interaction distances > 9 Å from the AIMD validation set. Structures with unit cells fulfilling this are thus not included not included.

Comparison of CPU hours and cell parameters for AIMD Validation Set

Table S1: The following table contains information on DFT single point calculations required to construct the single-particle grid (with grid size 0.4 Å and vdw scaling 0.7)for the respective structure along with number of cpu hours required to do so. This is compared with the number of AIMD steps (step size 2 ps) and cpu hours for corresponding structure. The grid calculations are run in parallel on a single node (32 cores) on the Tetralith HPC cluster (2x Intel Xeon Gold 6130) at The National Supercomputer Centre in Sweden. The AIMD calculations are run in paralell on single nodes (128 cores) on the Dardel HPC cluster (HPE Cray EX) at PDC Center for High Performance Computing in Sweden.

	**SP grids**		**AIMD**	
**Structure**	**grid points**	**cpu hours**	**MD steps**	**cpu hours**
$Li_{1.3}Al_{0.3}Ti_{1.7}(PO_4)_3$	2516	551	500’000	39’111
$Li_{10}GeP_2S_{12}$	1661	46	50’000	1’200
cubic$-Li_7La_3Zr_2O_{12}$	224	82	100’000	24’000
$Li_{12}Si_7$	3473	719	220’000	31’289
$Li_{13}Si_4$	1070	38	125’000	22’578
Li_2HN (no diff.)	285	12	200’000	44’900
$Li_3La_5Cl_{18}$	1302	199	250’000	31’111
Li_6PS_5Cl	206	40	400’000	8’888
$Li_7P_3S_{11}$	6086	219	100’000	7’111
$LiFe(CN)_3$	103	7	150’000	4’666
$LiMnO_2$	199	38	400’000	64’000
$LiNbO_3$	142	8	300’000	1’013
Li_3H_4Rh	296	8	100’000	6’133
$LiYF_2$	217	33	120’000	11’413

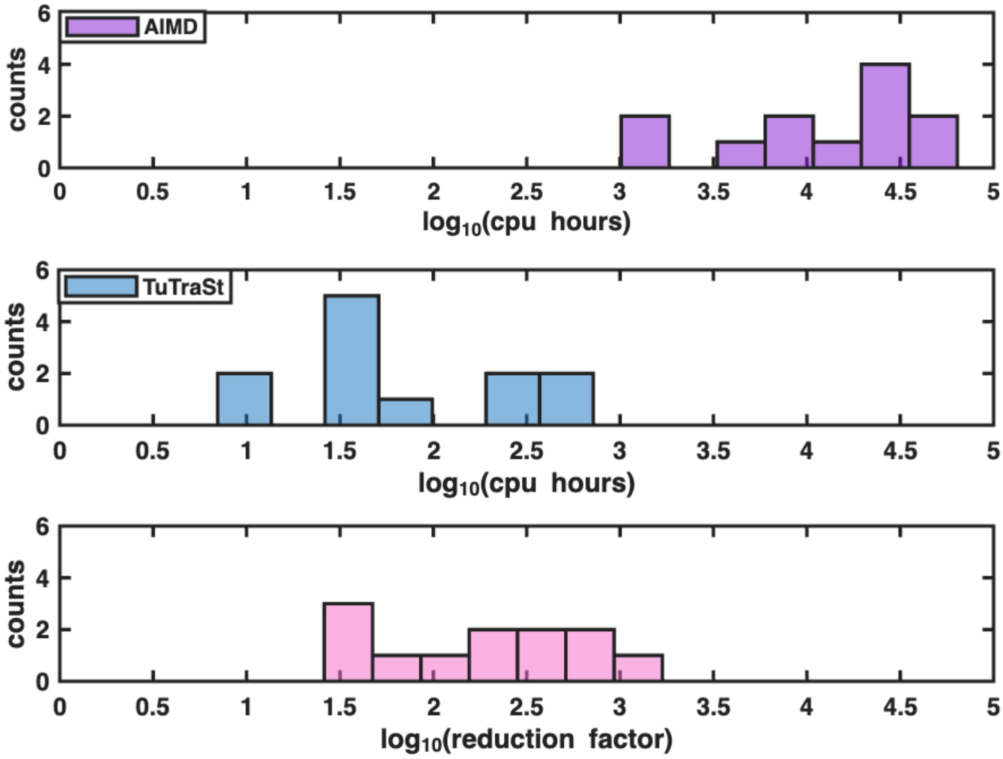

Figure S21: Comparison of total cpu hours required to computed the reported diffusion coefficients with AIMD (purple) vs the DFT-grid Ionic TuTrast method (blue) together with the corresponding reduction factors (pink). Numbers for Li_2HN are excluded in this comparison as the diffusive regime was not reached in the AIMD.

Table S2: Unit cell parameters used for single particle grid calculations and super cells used in AIMD calculations. Cell lengths (**a, b, c**) are given in Å and angles (α,β,γ) in degrees. Super cells are defined by multiples of the unit cells except Li_3H_4Rh where the grid and AIMD calculations are run on cell symmetries (same structure symmetry).

**Structure**	**grid unit cell (a,b,c,α,β,γ)**	**AIMD super cell**
$Li_{1.3}Al_{0.3}Ti_{1.7}$	$8.6, 8.6, 21.1, 90, 90, 120$	$1 \times 1 \times 1$
$Li_{10}GeP_2S_{12}$	$8.8, 8.8, 12.7, 90, 90, 90$	$1 \times 1 \times 1$
cubic$-Li_7La_3Zr_2O_{12\ 3}$	12.9,12.9,12.9,90,90,90	$1 \times 1 \times 1$
$Li_{12}Si_7$	$8.5, 14.3, 19.6, 90, 90, 90$	$1 \times 1 \times 1$
$Li_{13}Si_4$	$4.4, 7.9, 15.0, 90, 90, 90$	$3 \times 2 \times 1$
Li_2HN	$3.6, 4.9, 7.8, 90, 90, 90$	$3 \times 3 \times 2$
$Li_3La_5Cl_{18}$	$7.6, 7.6, 13.1, 90, 90120$	$2 \times 2 \times 1$
Li_6PS_5Cl	$10.3, 10.3, 10.3, 90, 90, 90$	$1 \times 1 \times 1$
$Li_7P_3S_{11}$	$6.2, 12.6, 12.7, 107.4, 103.5, 101.9$	$2 \times 1 \times 1$
$LiFe(CN)_3$	$9.9, 9.9, 9.9, 90, 90, 90$	$1 \times 1 \times 1$
$LiMnO_2$	$9.7, 9.7, 2.9, 90, 90, 90$	$1 \times 1 \times 3$
$LiNbO_3$	$8.0, 8.0, 8.0, 90, 90, 90$	$1 \times 1 \times 1$
Li_3H_4Rh	$3.8, 8.9, 8.8, 90, 90, 90$	$4.8, 4.8, 8.9, 90, 90, 134.6$
$LiYF_2$	$7.0, 7.0, 11.0, 90, 90, 90$	$1 \times 1 \times 1$

Li_6PS_5Cl PES Sampled by Different Models

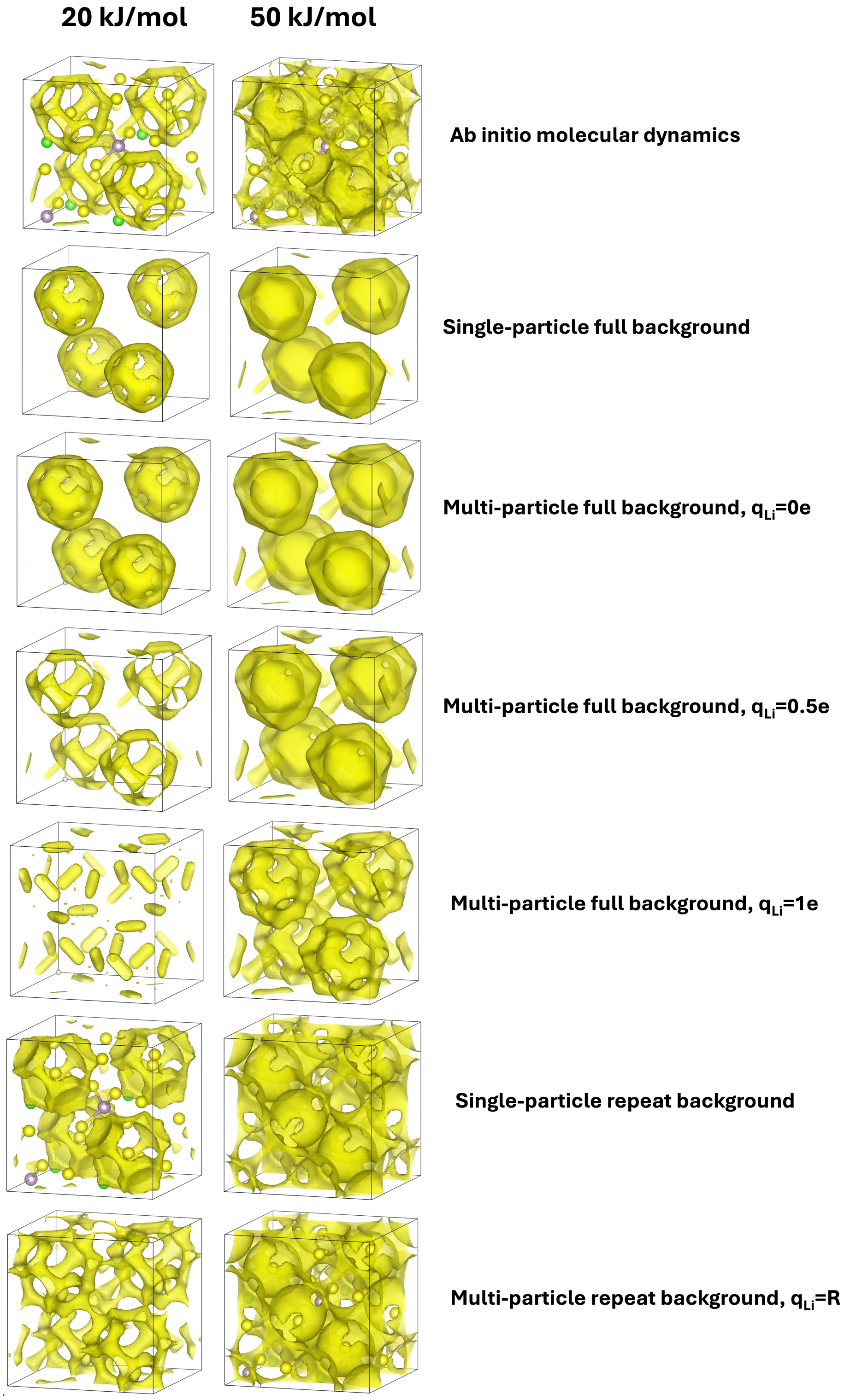

Figure S22: Potential Energy isosurfaces for Li_6PS_5Cl at 20 kJ/mol and 50 kJ/mol from different models. AIMD and multi-particle grids are constructed from trajectory output, while single-particle grids are constructed from the grid point sampling procedure presented in this work.

References

(S1) Dubbeldam, D.; Calero, S.; Ellis, D. E.; Snurr, R. Q. RASPA: molecular simulation software for adsorption and diffusion in flexible nanoporous materials. *Mol. Simul.* **2016**, *42*, 81–101, DOI: 10.1080/08927022.2015.1010082.
